# Modulating grapevine performance and hormonal dynamics under summer stress by the synergistic effects of kaolin and silicon

**DOI:** 10.3389/fpls.2025.1639169

**Published:** 2025-08-06

**Authors:** Sandra Pereira, Ana Monteiro, Miguel Baltazar, Carolina Maia, Sara Pereira, Manuel João Oliveira, Luís Pádua, Igor Gonçalves, Bruno Soares, Zélia Branco, Renata Moura, Damián Balfagón, José Moutinho-Pereira, Lia-Tânia Dinis

**Affiliations:** ^1^ Centre for the Research and Technology of Agro-Environmental and Biological Sciences (CITAB), University of Trás-os-Montes e Alto Douro (UTAD), Vila Real, Portugal; ^2^ Inov4Agro - Institute for Innovation, Capacity Building and Sustainability of Agri-Food Production, University of Trás-os-Montes e Alto Douro (UTAD), Vila Real, Portugal; ^3^ ADVID- Associação para o Desenvolvimento da Viticultura Duriense | CoLAB Vines & Wines, Parque de Ciência e Tecnologia de Vila Real – Régia Douro Park, Vila Real, Portugal; ^4^ Departmento de Biología, Bioquímica y Ciencias Naturales, Universitat Jaume I, Castellón de la Plana, Spain

**Keywords:** foliar application, grapevine resilience, heat and drought stress, kaolin, photosynthetic efficiency, secondary metabolites, silicon

## Abstract

**Introduction:**

Climate change is intensifying heat and drought stress in viticulture, negatively impacting yield and grape quality. High temperatures accelerate sugar accumulation and reduce organic acids, disrupting wine balance. Drought also lowers grapevine resilience by reducing stomatal conductance and photosynthetic efficiency, highlighting the need for sustainable strategies. This study evaluated the effects of foliar applications of kaolin (Kl) and silicon (Si) mixtures on grapevine physiology and fruit quality under summer stress.

**Methods:**

The experiment was conducted over two seasons (2023–2024) in a commercial vineyard (Quinta de Ventozelo, Douro Region) using the Touriga Franca variety. Treatments included a control and four formulations (MiKS 1 to 4), all with 2% Kl and Si ranging from 2% to 8%. Physiological measurements included gas exchange, chlorophyll a fluorescence, and leaf water potential. Biochemical analyses assessed pigments, sugars, proteins, phenols, flavonoids, *ortho*-diphenols, and leaf anatomy. Hormonal profiling (abscisic acid (ABA), indole-3-acetic acid (IAA), jasmonic acid (JA), and salicylic acid (SA)) was also performed.

**Results:**

Si and Kl treatments, particularly MiKS 3 and MiKS 4, significantly enhanced gas exchange parameters, water potential, and chlorophyll fluorescence under high-stress conditions. These treatments also increased chlorophyll, carotenoids, cuticular waxes, and cuticle thickness, contributing to improved plant vitality and stress resilience. Secondary metabolites such as *ortho*-diphenols were also enhanced. Hormonal profiling showed increased ABA and JA and decreased IAA and SA, suggesting strengthened stress signalling and defence responses.

**Discussion:**

Overall, Si and Kl mixtures effectively mitigated summer stress, improving grapevine physiological, biochemical, and anatomical responses under challenging climate conditions.

## Highlights

- Si and Kl foliar sprays improved vine resilience to summer stress.
- Higher Si doses enhanced photosynthesis and gas exchange at midday.
- Treatments increased flavonoids, phenols, and ortho-diphenols in leaves.
- Si and Kl improved water potential and leaf anatomical traits.
- Hormonal shifts supported improved stress response and growth balance.

## 1 Introduction

Global climate change has leading to rising temperatures and decreasing water availability, which pose significant challenges to producing high-quality wine grapes, especially in regions that are already warm and dry. When daily maximum temperatures exceed 35°C, commonly known as *“*hot days*”*, grapevines decline in essential physiological functions (Keller, 2020). This heat exposure compromises grape quality by reducing the main organic compounds, such as organic acids and anthocyanins, while increased sugar concentrations, increasing the alcohol content in wine. These imbalances are undesirable for winemakers who aim to produce balanced wine flavours (Gutiérrez-Gamboa et al., 2021; Suter et al., 2021). Water availability complicates this scenario, as the majority of vineyards remain unirrigated, exposing grapevines to the natural drought cycles. While moderate water stress can improve grape quality by promoting the synthesis of metabolites like anthocyanins and phenolics (Ju et al., 2019; Yang et al., 2020), severe and prolonged heat and drought events negatively impact grapevine growth, yield, and fruit metabolism, thus threatening both local production and the global distribution of viable winegrowing regions (Keller et al., 2016; Galat et al., 2019; Venios et al., 2020).

Research has extensively investigated grapevine responses to water stress (Flexas et al., 2002; Medrano et al., 2003; Dayer et al., 2013, 2016; Keller et al., 2016). However, relatively less attention has been on heat stress alone (Sadras and Soar, 2009; Soar et al., 2009; Carvalho et al., 2015) and even less studies examine the combined effects of heat and water stress on grapevines (Edwards et al., 2011; Sadras et al., 2012; Rocheta et al., 2014). Among the few studies examining this combined effect, findings have shown that grapevines experiencing water deficit are more susceptible to heat stress, exhibiting more severe declines in physiological responses such as stomatal conductance, pre-dawn leaf water potential, and net photosynthesis. These stresses can lead to significant leaf area loss, further impairing the grapevine*’*s capacity to tolerate long high temperature periods (Edwards et al., 2011). According to Sadras et al. (2012), water and heat stress together have a consistent effect on the plasticity of stomatal conductance. Water deficit reduces stomatal conductance under all environmental conditions, whereas elevated temperatures enhance both conductance and photosynthesis when conditions support a high rate of gas exchange.

These studies underscore the importance of management strategies that can mitigate the combined effects of drought and heat. Foliar applications of kaolin (Kl) and silicon (Si) have emerged as promising approaches to enhance grapevine resilience, reducing losses in quality and yield in the context of increasing climate variability. Indeed, these treatments work by reducing leaf temperature, minimizing water loss, and strengthening the plant*’*s natural defences, thereby decreasing the need for chemical treatments as well as supporting sustainable viticulture.

Kaolin (Al_2_Si_2_O_5_(OH)_4_), a white, non-toxic aluminosilicate clay, forms a reflective film when applied to leaves, deflecting harmful ultraviolet and infrared radiation while protecting photosystem II from excessive solar exposure (Shellie and King, 2012; Sharma et al., 2015). In grapevines, kaolin reduces leaf temperature and can improve grape quality by stimulating anthocyanin and flavonoid synthesis through activation of the phenylpropanoid and flavonoid pathways, while total soluble solids have shown a variation according to grape variety and time of application, with some studies reporting enhanced soluble solids following early applications, while others note greater effects with later treatments (Conde et al., 2016; Dinis et al., 2016; Dinis et al., 2018a; Kok and Bal, 2018; Luzio et al., 2021; Bernardo et al., 2022).

Silicon, one of Earth*’*s most abundant elements, is not essential for plant survival (Epstein, 1994) but it is widely recognized for enhancing plant resilience/adaptation to both biotic and abiotic stresses (Schabl et al., 2020). When applied through Si-based fertilizers, silicon improves grapevine tolerance by regulating pH, optimizing nutrient uptake, and inducing beneficial biochemical, physiological, and genetic adaptations that enhance stress resistance (Etesami and Jeong, 2018; Jain et al., 2017; Oliva et al., 2020; Rastogi et al., 2021; Pereira et al., 2024).

The combined application of kaolin and silicon in vineyard management provides a promising, low-impact approach to mitigating the effects of climate change on grapevine health and grape quality. The present study evaluates the effects of combining 2% kaolin mixed with various Si concentrations, ranging from 2 to 8%, on grapevine physiology, biochemistry, and grape quality. During the veraison and maturation periods in 2023 and 2024, an experimental study was conducted analysing predawn leaf water potential, gas exchange parameters (stomatal conductance, transpiration rate, net CO_2_ assimilation rate, intrinsic water use efficiency, and the Ci/Ca ratio), and chlorophyll a fluorescence (ϕPSII, qP, F_v_/F_m_, and NPQ). Leaves from different treatments during veraison and maturation were subjected to laboratory analyses, namely biochemical parameters (pigments, phenols, flavonoids, ortho-diphenols, sugars, and protein content) and histological parameters (total leaf thickness, upper and lower cuticle, upper and lower epidermis, palisade and spongy mesophyll cells, and mesophyll thickness).

By investigating these treatments, we aim to advance resilient viticultural practices that improve grapevine tolerance to climate variability, thereby contributing to the sustainability of wine production in climate-sensitive regions as the Douro Region.

## 2 Materials and methods

### 2.1 Climate conditions

**Figure 1:**
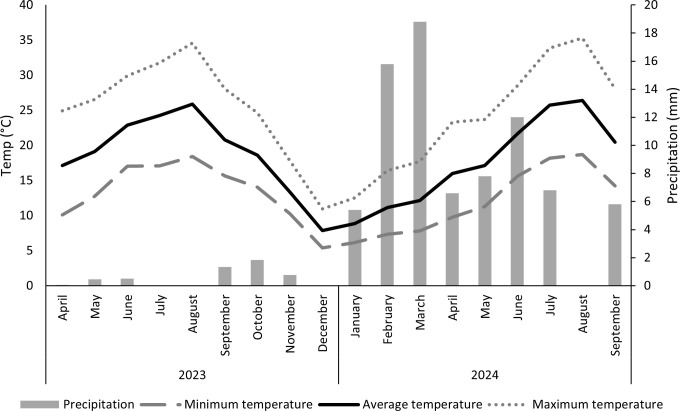
Monthly precipitation (mm) and air temperature (minimum, average and maximum) (ºC) from April 2023 to September 2024.

The experiment was conducted in a commercial vineyard *“*Quinta de Ventozelo*”* (41° 18.954´ N 8° 38.940´ W), located in Ervedosa do Douro, in the Cima Corgo sub-region, which has a moderate Mediterranean climate, with hot, dry summers and mild, wet winters, making it ideal for producing high-quality Port and Douro wines. Meteorological conditions (precipitation, and minimum, average and maximum temperatures) prevailing during the experimental period are presented in **Figure 1**. These data were collected by a weather station located within the vineyard.

Climate data from 2023 revealed a gradual increase in average temperature, rising from approximately 17°C in April to a maximum of 26°C in August, then decreasing to 8°C in December. Minimum temperatures followed a similar trend, starting at 10°C in April, rising to 18°C in August, and then dropping to 5°C in December. The maximum temperatures also demonstrated the same pattern, reaching a maximum at 35°C in August. The year 2023 was characterised by a below-average precipitation year, with a dry period during summer (Instituto Português do Mar e da Atmosfera (IPMA), 2024). In fact, the highest rainfall was recorded in September and October, though overall levels remained low, with a particularly dry period from July to August where rainfall was absent. This dry period, along with high temperatures, suggests potential water stress for the grapevines, influencing irrigation requirements and physiological responses.

In 2024, the average temperature began at approximately 9°C in January, gradually increased to 26°C in August, and then started to decline, reaching 20°C in September. The minimum temperature followed a similar trend, starting at 6°C in January, to a high 19°C in August, and decreasing to 14°C in September. The maximum temperature was around 13°C in January, 35°C in August, and then dropped to 28°C in September. Regarding precipitation, the highest levels were recorded in March, reaching approximately 19mm, while no rainfall was observed in August. Compared to 2023, the year 2024 was significantly wetter, with much higher precipitation levels.

### 2.2 Experimental design

The present study was carried out during two growing seasons: 2023 and 2024, in a vineyard planted in 2014 with the Touriga Franca grapevine variety, grafted onto 1103P rootstock. In the first year, four treatments were evaluated: untreated grapevines (control) and grapevines with foliar applications of three different silicon (Si) and kaolin (Kl) formulations. Each formulation contained 2% Kl, with varying Si concentrations ranging from 2% to 6%: MiKS 1- 2% Kl + 2% Si; MiKS 2- 2% Kl + 4% Si; MiKS 3- 2% Kl + 6% Si. The Si concentrations were based on a previous study (Dinis et al., 2024).

**Figure 2:**
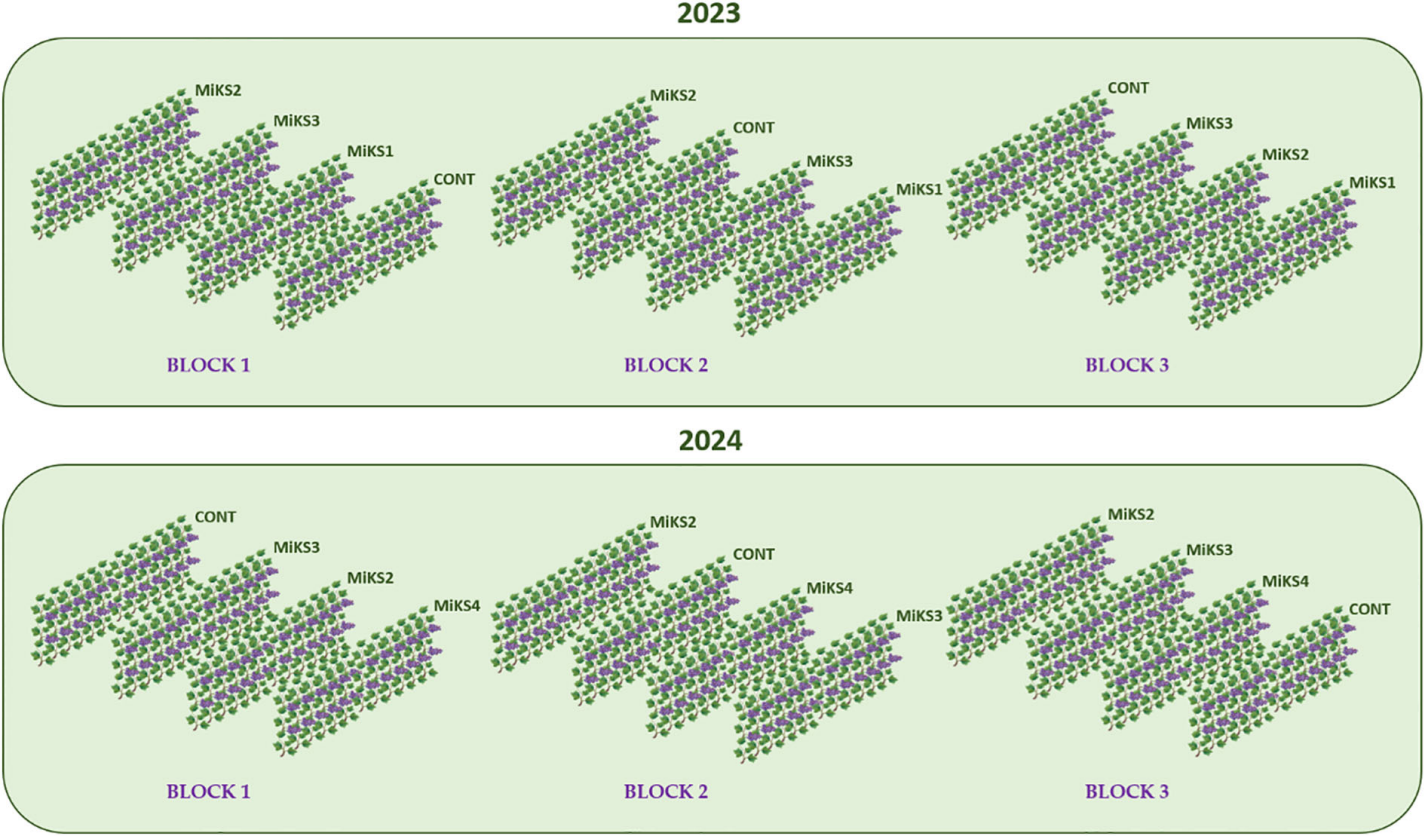
Experimental design of the field study conducted over two years (2023 and 2024), including the following treatments: untreated grapevines (control) and grapevines receiving foliar applications of four different silicon (Si) and kaolin (Kl) formulations. Each formulation contained 2% kaolin with varying silicon concentrations ranging from 2% to 8%: MiKS 1 – 2% Kl + 2% Si; MiKS 2 – 2% Kl + 4% Si; MiKS 3 – 2% Kl + 6% Si; MiS 4 – 2% Kl + 8% Si.

The experimental design consisted of three randomized blocks for each treatment, totalling 12 rows. In each row, applications were made to 15 grapevines. In the second year, based on the results obtained in 2023, the treatments were adjusted. Specifically, the MiKS1 treatment was discontinued, and instead, a higher concentration of Si was tested: MiKS 4, containing 2% Kl and 8% Si. The other treatments remained unchanged (**Figure 2**).

The different formulations were applied twice each year, with a two-week interval between applications, occurring in pre-veraison (mid-June and early July) when the summer stress begun.

### 2.3 Physiological parameters

#### 2.3.1 Gas exchange measurements

Leaf gas exchange measurements were performed on 3 plants per block, in a total of 9 measurements per treatment by using a portable LCpro + Infrared Gas Analyzer System (IRGA) (ADC BioScientific, Ltd., Hoddesdon, UK), with a 6.25 cm^2^ leaf chamber, operating in the open mode, on well-exposed leaves during the morning (09:00–10:30 a.m.) and solar noon (2:00–3:30 p.m.), in veraison (27 July 2023 and 18 July 2024) and maturation stages (24 August 2023 and 29 August 2024).

Net CO_2_ assimilation rate (*A*), stomatal conductance (*g_s_*), and transpiration rate (*E*) were estimated from gas exchange measurements, using the equations developed by Von Caemmerer and Farquhar (1981). Intrinsic water-use efficiency was calculated as the ratio of *A* to *gs* (*A/g_s_*) (Iacono et al., 1998) and the ratio Ci/Ca was also calculated.

#### 2.3.2 Chlorophyll a fluorescence measurement

The chlorophyll *a* fluorescence measurement was evaluated on the same leaves and stages used for gas exchange measurements (3 measurements per block, in a total of 9 per treatment). A pulse amplitude modulated fluorimeter was used (Mini-PAM, Photosynthesis Yield Analyzer; Walz, Effeltrich, Germany). The maximum quantum efficiency of photosystem II (*PSII*) was calculated as *F_v_/F_m_* = (*F_m_*−*F_0_*)/*F_m_* by measuring the fluorescence signal from a dark-adapted leaf when all reaction centres are open using a low-intensity pulsed measuring light source (*F_0_*) and during a pulse saturating light (0.7 s pulse of 15000 μmol·m^−2^·s^−1^ of white light) when all reactions centres are closed (*F_m_*). Leaves were dark-adapted for 30 min using dark-adapting leaf-clips for these measurements. Following *F_v_*/*F_m_* estimation, after a 20 s exposure to actinic light (1500 μmol·m^−2^·s^−1^), light-adapted steady-state fluorescence yield (*F_s_*) was averaged over 2.5 s, followed by exposure to saturating light (15000 μmol·m^−2^·s^−1^) for 0.7 s to establish *F_m_* ′. The sample was then shaded for 5s with a far-red light source to determine *F_0_* ′. From these measurements, several fluorescence attributes were calculated (Bilger and Schreiber, 1986; Genty et al., 1989): photochemical quenching (*qP*=(*F_m_* ′−*F_s_*)/(*F_m_* ′−*F_0_* ′)), non-photochemical quenching (*NPQ* = (*F_m_* − *F_m_* ′)/*F_m_*) and efficiency of electron transport as a measure of the quantum effective efficiency of *PSII* (*ΦPSII* = *ΔF*/*F_m_* **′** = (*F_m_* **′** - *F_s_*)/*F_m_* **′**). The apparent electron transport rate (Marinari et al., 2007) was estimated as *ETR* = (*ΔF*/*F_m_* **′**) x *PPFD* x 0.5 x 0.84, where PPFD is the photosynthetic photon flux density incident on the leaf, 0.5 is the factor that assumes equal distribution of energy between the two photosystems, and the leaf absorbance used was 0.84 because is the most common value for C3 plants (Bilger and Schreiber, 1986).

#### 2.3.3 Predawn leaf water potential

Predawn leaf water potential (Ψ) was determined with a pressure chamber (model PMS 600, Albany, USA) (Scholander et al., 1965) in veraison and maturation stages. Measurements were performed on nine fully expanded leaves per treatment at predawn (3 measurements per block, in a total of 9 leaves per treatment).

Aerial surveys were conducted only in 2024 to assess canopy reflectance and temperature during veraison. Two unmanned aerial vehicles (UAVs) were used: the Mavic 3T (DJI, Shenzhen, China) and the P4 Multispectral (DJI, Shenzhen, China). The Mavic 3T was used to acquire high-resolution RGB (12 MP) and thermal infrared (TIR) imagery using an uncooled VOx microbolometer with a pixel pitch of 12 µm. UAV flights were performed on 18 July (veraison stage) at three time points (09:30, 11:55, and 13:30 local time) corresponding to early morning, mid-morning and solar noon, following the same flight plan: 40 m height above the ground level, 90% imagery longitudinal overlap, and 80% imagery lateral overlap.

The P4 Multispectral UAV acquires data across five spectral bands using monochrome sensors for blue (450 ± 16 nm), green (560 ± 16 nm), red (650 ± 16 nm), red edge (730 ± 16 nm), and near-infrared (NIR, 840 ± 26 nm). An integrated spectral sunlight sensor recorded incoming irradiance to enable radiometric correction. A single flight survey was conducted at 12:30 at 50 m height form the take-off point with 80% longitudinal and 70% lateral imagery overlap. Prior to the flight, images of a reflectance target were captured to enable radiometric calibration of the multispectral data.

During the TIR flights, dry and wet reference temperatures were collected using a handheld infrared thermometer on sun-exposed leaves. For the dry reference, petroleum jelly was applied to the abaxial leaf surface to occlude stomata, to minimize transpiration and increasing leaf surface temperature. The wet reference was obtained by applying water to similar leaf surfaces, with temperature measured immediately to capture the evaporative cooling effect.

UAV imagery was processed in Pix4Dmapper (Pix4D SA, Lausanne, Switzerland). Separate projects were created for each flight, with the spatial alignment across datasets being ensured. The outputs included RGB orthophoto mosaics, a digital surface model (DSM), a digital terrain model (DTM), land surface temperature (LST) rasters from TIR imagery, and radiometrically calibrated reflectance rasters for each multispectral band.

The RGB data had a spatial resolution of 0.009 m, TIR data 0.0365 m, and multispectral data 0.0278 m.

Subsequently, the computed raster products were imported into QGIS for further analysis. For each treatment and replicate, a 10-m section of vineyard row was selected. From the central part of each row, a central line was estimated and buffered into a 0.2 m wide polygon, then segmented into 20 sub-polygons (0.5 m × 0.2 m). These were used to extract canopy temperature data. The median temperature of each polygon was calculated to reduce the influence of outliers, such as soil pixels or shaded areas. Polygons with missing plants or low vegetative vigour were excluded.

The empirical crop water stress index (CWSI) (Idso et al., 1981) was calculated using the extracted temperature data and temperature dry and wet references. Mean CWSI values, computed from the polygon medians within each replicate, were used for statistical analysis as well as the median temperature in each performed flight.

For spectral reflectance analysis, a canopy surface model (CSM) was calculated from the subtraction of the DTM altitude values to the DSM. Pixels with a CSM height above 0.8 m were considered canopy, and their mean reflectance values across each of the five spectral bands were extracted.

### 2.4 Leaf biochemistry

Each year, at both the veraison and maturation stages, three leaves per block (totalling nine leaves per treatment) were randomly collected and immediately preserved in liquid nitrogen. In the laboratory, the leaves were ground using liquid nitrogen and then stored at -80°C until analysis. All leaf analyses are expressed on a fresh weight basis.

#### 2.4.1 Photosynthetic pigments

To quantify the photosynthetic pigments, 10 mg of fresh leaf tissue (FW) was combined with 4 ml of 80% acetone. The mixture was then centrifuged at 1,753 x *g* for 10 minutes at 4°C. Post-centrifugation, absorbance readings were taken at 663 nm, 645 nm, and 470 nm using a SPECTRUM star Nano spectrophotometer (BMG Labtech GmbH, Germany). Given the light and temperature sensitivity of these pigments, all extraction and quantification steps were performed on ice and shielded from light. Pigment concentrations were calculated according to Arnon (1949) and Lichtenthaler (1987), with results expressed in mg g^-1^ FW.

#### 2.4.2 Soluble sugars

To quantify soluble sugars, 10 mg of each sample was extracted in 5 ml of 80% ethanol. The extracts were homogenized and heated at 80°C in a water bath for 60 minutes, then centrifuged at 15,777 *g* to collect the supernatant. Next, 250 μL of each sample was mixed with 750 μL of anthrone reagent. The mixture was chilled at 4°C for 10 minutes, followed by incubation at 100°C for 20 minutes. Absorbance was measured at 625 nm (Leyva et al., 2008). A glucose standard curve was used for quantification, and results were expressed as mg g^-1^ FW.

#### 2.4.3 Soluble proteins

Soluble proteins were extracted using a phosphate-based extraction buffer (pH 7.5; Fisher Scientific, Loughborough, UK) supplemented with EDTA (ethylenediaminetetraacetic acid; Panreac, Barcelona, Spain). The working solution contained this extraction buffer along with PMSF (phenylmethanesulfonyl fluoride; Panreac, Barcelona, Spain) and PVP (polyvinylpyrrolidone; Sigma, St. Louis, MO, USA). Protein quantification followed the Bradford method (Bradford, 1976), with absorbance measured at 595 nm. Soluble protein content was expressed as milligrams of bovine serum albumin equivalents per gram of fresh weight (mg BSAE g^-1^ FW).

#### 2.4.4 Secondary metabolites

To determine the secondary metabolites in the leaves, a methanolic extract at a concentration of 4 mg ml^-1^ was prepared and used for the following quantifications.

Total phenols were quantified using the Folin–Ciocalteu method, with absorbance measured at 725 nm (Rodrigues et al., 2015). Results were expressed in milligrams of gallic acid equivalents per gram of fresh weight (mg GAE g^-1^ FW).

Total flavonoid content in the extracts was quantified using the aluminium chloride (AlCl_3_) complex method, with absorbance measured at 510 nm, as described by Rodrigues et al. (2015). Results were expressed as milligrams of catechin equivalents per gram of fresh weight (mg CAE g^-1^ FW).

*Ortho*-diphenols were measured by reading the absorbance at 370 nm, following the method described by Granato et al. (2015). A gallic acid standard curve was used for calibration, and results were expressed as milligrams per gram of fresh weight (mg g^-1^ FW).

### 2.5 Phytohormones

The contents of abscisic acid (ABA), indole-3-acetic acid (IAA), jasmonic acid (JA), and salicylic acid (SA) were determined using high-performance liquid chromatography coupled with a triple quadrupole mass spectrometer (Micromass^®^, Manchester, UK) via an orthogonal Z-spray electrospray ion source (Durgbanshi et al., 2005). Lyophilized samples (20 mg) of leaves were extracted in 2.0 mL of distilled water using a mill ball apparatus (MillMix20, Domel, Železniki, Slovenia). [²H_6_]-ABA, [²H_5_]-IAA, dihydrojasmonic acid (DHJA) and [¹³C_6_]-SA (Sigma-Aldrich, USA) were used as internal standards.

After centrifugation at 10,000 × g, the supernatants were collected, and the pH was adjusted to 2.8–3.2 with 30% acetic acid. Extracts were partitioned twice with diethyl ether, and the resulting supernatants were evaporated under vacuum using a centrifuge concentrator (Speed Vac, Jouan, Saint Herblain Cedex, France) at room temperature. The dry residue was resuspended in 500 μL of a 9:1 water:methanol solution, filtered through 0.22 μm PTFE filters, and directly injected into an ultra-performance liquid chromatography system (Waters™ Acquity SDS, Waters Corporation, Milford, MA, USA) interfaced with a TQD triple quadrupole mass spectrometer (Micromass^®^ Ltd., Manchester, UK).

Chromatographic separation was performed on a reversed-phase C18 column (Gravity, 50 × 2.1 mm, 1.8 μm particle size; Macherey-Nagel GmbH, Germany) using a methanol:water gradient supplemented with 0.1% acetic acid at a flow rate of 300 μL min^-1^. The aqueous phase was maintained at 90% for the first 2 minutes, decreased to 10% over 6 minutes, then increased back to 90% by 7 minutes, and held constant until the end of the 8-minute run.

Mass spectrometry was conducted in multiple reaction monitoring mode using nitrogen as the drying and nebulizer gas (cone gas flow: 250 L h^-1^; desolvation flow: 1200 L h^-1^) and argon as the collision gas. Cone voltage and collision energies were set according to Durgbanshi et al. (2005), with minor modifications. Data processing was carried out using MassLynx™ v4.1 software, and phytohormone concentrations (ng g^-1^ DW) were determined by interpolating the response ratios of the phytohormones to their internal standards against a calibration curve prepared with commercial ABA, IAA, JA, and SA standards.

### 2.6 Histological parameters

Anatomical tissue measurements were conducted on nine mature leaves per treatment, with three leaves collected from each of the three blocks. Leaf samples were collected from the central region to minimize variability in thickness. After fixation in formalin aceto-alcohol for 24 hours, the samples underwent dehydration, clearing, and paraffin embedding. Transverse sections (4 μm thick) were then prepared using a rotary microtome (Leica RM 2135, Germany). These sections were mounted on slides and stained with 0.1% toluidine blue. Tissue thickness was measured using an inverted optical microscope (Olympus IX51, Olympus Corporation, Tokyo, Japan) and analysed with Digimizer image analysis software (MedCalc Software, Ostend, Belgium).

### 2.7 Statistical analyses

Data analysis was performed using the SPSS 20.0 software (SPSS Software, Chicago, IL, USA). After testing for analysis of variance (ANOVA) assumptions, statistical differences among treatments within each developmental stage and year were evaluated by one-way factorial ANOVA, followed by the *post hoc* Tukey test. Different letters represent significant differences (*P*< 0.05) among the applied formulations.

## 3 Results

The results of this study highlight the significant impact of combining Kl and Si on grapevine physiology, biochemical composition, and anatomical adaptations under heat and drought stress conditions. The differential effects observed across treatments indicate that Si and Kl formulations play a critical role in enhancing plant resilience by modulating physiological responses, optimizing water use, improving photosynthetic efficiency, and strengthening biochemical defences.

### 3.1 Kaolin – silicon mixtures boost physiological responses in grapevines under summer stress

The gas exchange and chlorophyll *a* fluorescence parameters of treated plants with various mixtures of Kl and Si foliar applications were analysed during the morning and solar noon periods at the veraison and maturation phenological stages in both 2023 and 2024. The results are presented in **Tables 1**, **2**, respectively.

**Table 1:** Gas exchange parameters (mean±SD), namely transpiration rate (*E*, mmol m^-2^ s^-1^), stomatal conductance (*gs*, mmol m^-2^ s^-1^), intercellular carbon (*Ci*, µmol mol^-1^), net CO_2_ assimilation rate (*A*, μmol m^-2^ s^-1^), intrinsic water use efficiency (*A/gs*, µmol mol^-1^) and intercellular/atmospheric CO_2_ concentration ratio (*Ci/Ca*), in veraison and maturation of 2023 and 2024 (morning and solar noon) in Touriga Franca leaves subjected to combined application of Kl (2%) and Si (2 to 8%).

Year	Phenological stages	Time	Treatment	*E* (mmol.m^-2^.s^-1^)	*gs* (mmol.m^-2^.s^-1^)	*A* (μmol.m^-2^.s^-1^)	*A/gs* (µmol.mol^-1^)	*Ci/Ca*
**2023**	**Veraison**	**Morning**	**Control**	2.72 ± 0.30	133.7 ± 10.4 ab	11.2 ± 1.2	81.4 ± 2.9	0.655 ± 0.059
			**Miks 1 (Kl_2% + Si_2%)**	2.46 ± 0.31	126.2 ± 18.7 ab	10.2 ± 1.6	84.5 ± 7.2	0.599 ± 0.039
			**Miks 2 (Kl_2% + Si_4%)**	2.65 ± 0.67	158.2 ± 20.4 b	12.5 ± 1.2	79.1 ± 3.4	0.624 ± 0.059
			**Miks 3 (Kl_2% + Si_6%)**	2.14 ± 0.33	110.0 ± 11.5 a	10.9 ± 2.1	85.2 ± 2.6	0.652 ± 0.036
			***P* value**	0.236	0.008	0.229	0.287	0.199
		**Solar noon**	**Control**	2.95 ± 0.62 b	123.2 ± 22.6 b	8.4 ± 2.0 a	79.2 ± 8.3	0.641 ± 0.047
			**Miks 1 (Kl_2% + Si_2%)**	1.63 ± 0.24 a	66.8 ± 12.3 a	6.0 ± 1.0 a	89.0 ± 6.9	0.618 ± 0.030
			**Miks 2 (Kl_2% + Si_4%)**	2.46 ± 0.78 ab	132.7 ± 13.9 b	11.3 ± 1.3 b	88.3 ± 13.4	0.603 ± 0.019
			**Miks 3 (Kl_2% + Si_6%)**	2.34 ± 0.72 ab	85.8 ± 17.9 a	7.5 ± 1.6 a	86.7 ± 4.9	0.627 ± 0.053
			***P* value**	0.034	<0.001	<0.001	0.452	0.395
	**Maturation**	**Morning**	**Control**	2.46 ± 0.63 c	51.2 ± 6.4 b	3.6 ± 1.4 ab	90.9 ± 6.0	0.626 ± 0.083
			**Miks 1 (Kl_2% + Si_2%)**	0.70 ± 0.27 a	24.1 ± 6.2 a	2.4 ± 0.5 a	80.7 ± 6.6	0.625 ± 0.046
			**Miks 2 (Kl_2% + Si_4%)**	1.49 ± 0.27 b	50.2 ± 6.3 b	5.0 ± 1.0 b	90.4 ± 5.7	0.591 ± 0.029
			**Miks 3 (Kl_2% + Si_6%)**	0.60 ± 0.14 a	14.5 ± 3.2 a	1.8 ± 0.7 a	87.6 ± 6.6	0.619 ± 0.021
			***P* value**	<0.001	<0.001	0.003	0.128	0.639
		**Solar noon**	**Control**	0.47 ± 0.14 a	9.2 ± 2.8 ab	2.3 ± 0.9 a	155.3 ± 32.7	0.283 ± 0.054 a
			**Miks 1 (Kl_2% + Si_2%)**	1.01 ± 0.36 ab	21.2 ± 8.1 ab	1.3 ± 0.2 a	101.7 ± 47.3	0.648 ± 0.036 b
			**Miks 2 (Kl_2% + Si_4%)**	1.50 ± 0.47 b	24.1 ± 9.8 b	3.9 ± 0.8 b	138.6 ± 40.7	0.527 ± 0.153 ab
			**Miks 3 (Kl_2% + Si_6%)**	0.30 ± 0.14 a	6.0 ± 2.7 a	1.3 ± 0.2 a	151.3 ± 21.7	0.365 ± 0.053 a
			***P* value**	0.002	0.012	<0.001	0.264	0.007
**2024**	**Veraison**	**Morning**	**Control**	2.59 ± 0.85 a	73.0 ± 10.9 a	6.6 ± 1.2 a	90.2 ± 12.6	0.583 ± 0.047 ab
			**Miks 2 (Kl_2% + Si_4%)**	4.28 ± 0.46 b	74.2 ± 10.9 a	11.4 ± 1.4 c	114.1 ± 27.0	0.530 ± 0.048 a
			**Miks 3 (Kl_2% + Si_6%)**	4.05 ± 0.42 b	129.7 ± 26.4 b	9.3 ± 1.0 b	113.2 ± 22.1	0.625 ± 0.085 b
			**Miks 4 (Kl_2% + Si_8%)**	4.87 ± 0.96 b	120.5 ± 16.6 b	11.7 ± 1.4 c	102.4 ± 6.0	0.542 ± 0.045 a
			***P* value**	<0.001	<0.001	<0.001	0.322	0.009
		**Solar noon**	**Control**	0.81 ± 0.35 a	15.0 ± 5.3 a	2.5 ± 0.9 a	103.4 ± 16.2 a	0.504 ± 0.121
			**Miks 2 (Kl_2% + Si_4%)**	2.08 ± 0.46 b	50.0 ± 9.7 b	5.7 ± 1.4 b	125.5 ± 13.5 ab	0.510 ± 0.073
			**Miks 3 (Kl_2% + Si_6%)**	2.08 ± 0.55 b	48.6 ± 11.1 b	5.4 ± 0.9 b	161.4 ± 38.7 b	0.525 ± 0.094
			**Miks 4 (Kl_2% + Si_8%)**	2.39 ± 0.88 b	63.4 ± 18.8 b	9.3 ± 2.8 c	216.9 ± 44.9 c	0.440 ± 0.125
			***P* value**	<0.001	<0.001	<0.001	<0.001	0.484
	**Maturation**	**Morning**	**Control**	1.16 ± 0.33 a	94.1 ± 14.1 ab	5.0 ± 1.2 a	83.7 ± 9.8	0.618 ± 0.098
			**Miks 2 (Kl_2% + Si_4%)**	2.00 ± 0.44 b	86.1 ± 14.3 a	8.1 ± 0.8 b	85.9 ± 5.0	0.626 ± 0.052
			**Miks 3 (Kl_2% + Si_6%)**	2.53 ± 0.41 c	122.1 ± 11.9 b	9.9 ± 1.2 c	87.9 ± 6.2	0.599 ± 0.034
			**Miks 4 (Kl_2% + Si_8%)**	2.30 ± 0.20 bc	101.5 ± 34.8 b	9.1 ± 1.2 bc	86.8 ± 12.6	0.615 ± 0.040
			***P* value**	<0.001	0.007	<0.001	0.233	0.776
		**Solar noon**	**Control**	1.03 ± 0.35 a	25.6 ± 4.8	1.3 ± 0.4	59.2 ± 7.4 a	0.677 ± 0.105
			**Miks 2 (Kl_2% + Si_4%)**	1.38 ± 0.27 b	27.8 ± 2.3	1.8 ± 0.5	78.9 ± 16.2 ab	0.707 ± 0.095
			**Miks 3 (Kl_2% + Si_6%)**	1.37 ± 0.34 b	27.2 ± 3.3	2.1 ± 0.7	119.7 ± 29.3 bc	0.585 ± 0.085
			**Miks 4 (Kl_2% + Si_8%)**	1.38 ± 0.40 b	24.4 ± 3.6	2.1 ± 0.7	136.8 ± 32.1 c	0.597 ± 0.077
			***P* value**	0.025	0.519	0.909	<0.001	0.071

Different letters represent significant differences between the treatments at the same time of day and within the same phenological stage. The absence of letters indicates that there are no significant differences between treatments. n = 9.

**Table 2:** Chlorophyll *a* fluorescence parameters (mean ± SD): effective efficiency of PSII (*ΦPSII*), photochemical (*qP*), maximum quantum efficiency of photosystem II (*Fv/Fm*), and non-photochemical (*NPQ*) quenching, in veraison and maturation of 2023 and 2024 (morning and solar noon) in Touriga Franca leaves subjected to combined application of Kl (2%) and Si (2 to 8%).

Year	Phenological stages	Time	Treatment	*ΦPSII*	*Qp*	*Fv/Fm*	*NPQ*
2023	**Veraison**	**Morning**	**Control**	0.204 ± 0.083	0.581 ± 0.084	0.800 ± 0.039	8.26 ± 0.55 b
			**Miks 1 (Kl_2% + Si_2%)**	0.150 ± 0.041	0.571 ± 0.084	0.841 ± 0.041	7.61 ± 2.26 b
			**Miks 2 (Kl_2% + Si_4%)**	0.205 ± 0.048	0.639 ± 0.080	0.812 ± 0.075	2.49 ± 0.34 a
			**Miks 3 (Kl_2% + Si_6%)**	0.207 ± 0.037	0.593 ± 0.082	0.839 ± 0.052	8.06 ± 0.88 b
			***P* value**	0.243	0.522	0.487	<0.001
		**Solar noon**	**Control**	0.159 ± 0.033	0.613 ± 0.094	0.800 ± 0.046	3.27 ± 0.36
			**Miks 1 (Kl_2% + Si_2%)**	0.127 ± 0.036	0.588 ± 0.025	0.828 ± 0.025	2.94 ± 0.51
			**Miks 2 (Kl_2% + Si_4%)**	0.117 ± 0.035	0.650 ± 0.079	0.802 ± 0.041	2.98 ± 0.55
			**Miks 3 (Kl_2% + Si_6%)**	0.138 ± 0.036	0.648 ± 0.086	0.831 ± 0.042	3.58 ± 0.58
			***P* value**	0.307	0.502	0.393	0.170
	**Maturation**	**Morning**	**Control**	0.100 ± 0.018	0.634 ± 0.039	0.737 ± 0.028	4.17 ± 0.43
			**Miks 1 (Kl_2% + Si_2%)**	0.108 ± 0.029	0.560 ± 0.095	0.723 ± 0.065	3.85 ± 0.94
			**Miks 2 (Kl_2% + Si_4%)**	0.106 ± 0.035	0.634 ± 0.063	0.744 ± 0.053	4.21 ± 0.66
			**Miks 3 (Kl_2% + Si_6%)**	0.145 ± 0.034	0.618 ± 0.070	0.738 ± 0.073	3.73 ± 0.74
			***P* value**	0.142	0.261	0.934	0.684
		**Solar noon**	**Control**	0.064 ± 0.033	0.475 ± 0.071	0.736 ± 0.099	3.06 ± 0.79 a
			**Miks 1 (Kl_2% + Si_2%)**	0.068 ± 0.022	0.576 ± 0.120	0.639 ± 0.097	4.62 ± 1.20 b
			**Miks 2 (Kl_2% + Si_4%)**	0.063 ± 0.021	0.612 ± 0.126	0.777 ± 0.074	4.17 ± 0.43 ab
			**Miks 3 (Kl_2% + Si_6%)**	0.087 ± 0.028	0.537 ± 0.082	0.772 ± 0.106	4.81 ± 0.47 b
			***P* value**	0.445	0.280	0.102	0.010
2024	**Veraison**	**Morning**	**Control**	0.071 ± 0.048	0.337 ± 0.082	0.866 ± 0.066	5.42 ± 1.17
			**Miks 2 (Kl_2% + Si_4%)**	0.130 ± 0.046	0.443 ± 0.066	0.890 ± 0.053	6.30 ± 0.71
			**Miks 3 (Kl_2% + Si_6%)**	0.090 ± 0.051	0.381 ± 0.079	0.861 ± 0.100	6.52 ± 1.01
			**Miks 4 (Kl_2% + Si_8%)**	0.108 ± 0.045	0.398 ± 0.038	0.908 ± 0.049	6.53 ± 1.33
			***P* value**	0.071	0.182	0.307	0.087
		**Solar noon**	**Control**	0.088 ± 0.023 a	0.357 ± 0.064	0.875 ± 0.053 a	6.35 ± 1.03 a
			**Miks 2 (Kl_2% + Si_4%)**	0.116 ± 0.030 ab	0.376 ± 0.068	0.928 ± 0.030 b	6.09 ± 0.81 a
			**Miks 3 (Kl_2% + Si_6%)**	0.131 ± 0.024 b	0.439 ± 0.110	0.924 ± 0.048 b	7.79 ± 1.05 b
			**Miks 4 (Kl_2% + Si_8%)**	0.107 ± 0.029 ab	0.408 ± 0.066	0.880 ± 0.057 a	8.55 ± 0.92 b
			***P* value**	0.049	0.229	0.031	<0.001
	**Maturation**	**Morning**	**Control**	0.103 ± 0.013 a	0.360 ± 0.046	0.818 ± 0.053	4.31 ± 0.92 a
			**Miks 2 (Kl_2% + Si_4%)**	0.126 ± 0.033 ab	0.423 ± 0.044	0.795 ± 0.052	5.43 ± 0.62 b
			**Miks 3 (Kl_2% + Si_6%)**	0.144 ± 0.031 b	0.371 ± 0.059	0.811 ± 0.027	4.27 ± 0.68 a
			**Miks 4 (Kl_2% + Si_8%)**	0.146 ± 0.026 b	0.378 ± 0.074	0.790 ± 0.042	4.54 ± 0.74 ab
			***P* value**	0.007	0.174	0.507	0.040
		**Solar noon**	**Control**	0.083 ± 0.014	0.330 ± 0.051	0.784 ± 0.042	6.08 ± 1.15
			**Miks 2 (Kl_2% + Si_4%)**	0.096 ± 0.026	0.329 ± 0.071	0.771 ± 0.046	6.88 ± 1.20
			**Miks 3 (Kl_2% + Si_6%)**	0.102 ± 0.043	0.330 ± 0.050	0.730 ± 0.058	6.22 ± 1.08
			**Miks 4 (Kl_2% + Si_8%)**	0.101 ± 0.035	0.331 ± 0.052	0.713 ± 0.089	5.57 ± 0.84
			***P* value**	0.647	1.000	0.710	0.158

Different letters represent significant differences between the treatments at the same time of day and within the same phenological stage. The absence of letters indicates that there are no significant differences between treatments. n = 9.

**Figure 3:**
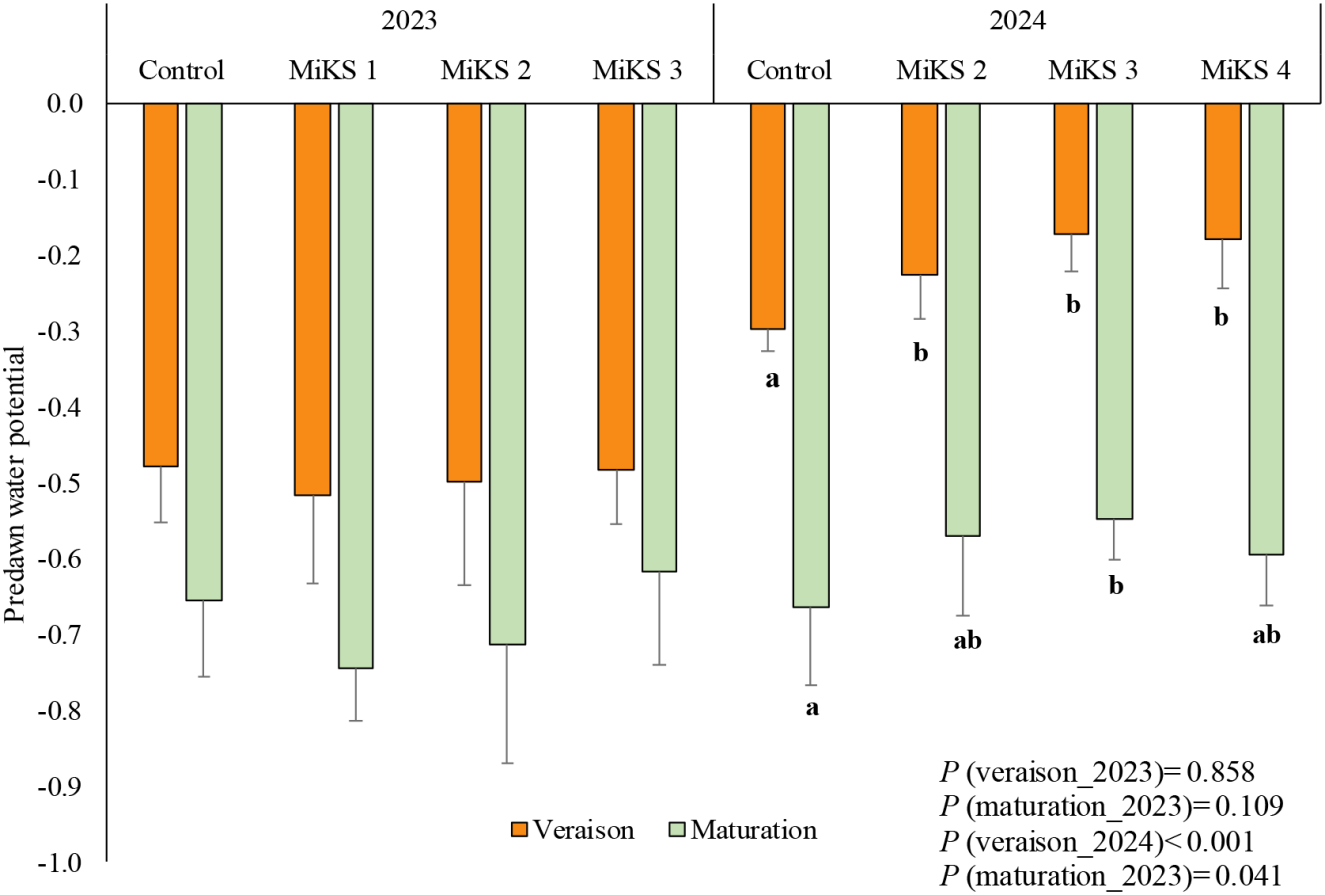
Predawn leaf water potential (mean ± SD), in veraison and maturation of 2023 and 2024, in Touriga Franca leaves subjected to combined application of Kl (2%) and Si (2 to 8%). Different letters represent significant differences between the treatments within the same phenological stage. The absence of letters indicates that there are no significant differences between treatments. n = 9.

Additionally, the predawn water potential was assessed at both the veraison and maturation stages in 2023 and 2024, as shown in **Figure 3**.

In the veraison of 2023, during the morning measurements, significant differences were observed only in *gs* (**Table 1**), where MiKS 2-treated plants had significantly higher *gs* than those treated with MiKS 3, with an increase of 43.8%. For the solar noon period, MiKS 1-treated plants showed the lowest values for *gs*, *E*, and *A*. Compared to MiKS 1, untreated plants exhibited significantly higher *E* and *gs*, with increases of 81.0% and 84.4%, respectively. MiKS 2-treated plants displayed significantly higher *gs* and *A* values than both MiKS 1 (98.7% and 90.2% increases, respectively) and MiKS 3 (54.7% and 51.7% increases, respectively). Additionally, MiKS 2-treated plants showed significantly higher *A* values compared to the control, with an increase of 35.0%. Regarding chlorophyll *a* fluorescence, during the same period, *NPQ* showed the only significant difference, presented MiKS 2-treated plants 69.9%, 67.3% and 69.1% lower values compared to control, MiKS 1 and MiKS 3 plants.

In maturation stage of 2023, during the morning measurements, control plants exhibited significantly higher *E* and *gs* values than MiKS 1-treated plants (252.4% and 112.4% increases, respectively) and MiKS 3-treated plants (310.7% and 253.1% increases, respectively). MiKS 2-treated plants also exhibited significantly higher *gs* and *E* compared to both MiKS 1 and MiKS 3, with increases of 108.3% and 246.2% for *gs*, and 113.5% and 148.7% for *E*, respectively. Similar results were observed for *A*, with MiKS 2-treated plants having significantly higher values than MiKS 1 and MiKS 3-treated plants, with increases of 112.3% and 172.7%, respectively. During the solar noon period, MiKS 2-treated plants exhibited the highest values for *gs*, *E*, and *A*, with increases of 305.0% (*gs*), 398.3% (*E*), and 205.5% (*A*) compared to MiKS 3-treated plants, which recorded the lowest values. The highest *Ci/Ca* ratio was observed in MiKS 1-treated plants, with a 129.0% increase over the control and a 77.5% increase compared to MiKS 3-treated plants. Regarding chlorophyll *a* fluorescence, on the solar noon period in the maturation of 2023, control plants had the lowest *NPQ* values, with reductions of 33.8% and 36.4% compared to MiKS 1- and MiKS 3-treated plants, respectively.

Regarding predawn leaf water potential, no significant differences were observed in 2023 in either veraison or maturation (**Figure 3**).

Regarding 2024, during veraison in the morning, plants treated with the mixtures generally showed significantly higher *gs*, *E*, and *A* values. MiKS 3-treated plants had the highest *Ci/Ca* ratio, which was 17.9% higher than MiKS 2 and 15.3% higher than MiKS 4. In the solar noon period, MiKS 4-treated plants had the highest values of *gs*, *E*, *A*, and *A/gs*, while control plants recorded the lowest values. In fact, in comparison to the control, MiKS 4-treated plants increased 322.7% in *gs*, 194.0% in *E*, 272.7% in *A*, and 109.8% in *A/gs*. However, overall, all formulations resulted in increased values of *E*, *gs*, *A*, and *A/gs* during this phenological stage.

Regarding chlorophyll *a* fluorescence during veraison in 2024, significant differences were observed at solar noon. MiKS 3-treated plants had *ΦPSII* values that were 48.9% higher than untreated plants. In terms of *F_v_/F_m_*, MiKS 2- and MiKS 3-treated plants displayed significantly higher values compared to control and MiKS 4, with increases ranging from 5.0% to 6.0%. The *NPQ* was significantly higher in treated plants with the higher doses of Si (MiKS 3 and MiKS 4) compared to both control and MiKS 2-treated plants, with increases ranging from 22.7% to 40.4%.

During maturation in 2024, in the morning period, MiKS 3 and MiKS 4-treated plants exhibited significantly higher *gs* values compared to MiKS 2, with increases of 41.8% and 17.9%, respectively. Additionally, MiKS 3-treated plants also had higher *E* (118.1%) and *A* (100.2%) values than control ones. In the solar noon period, all formulations induced an *E* increase compared to control, with increases ranging from 33.0% to 34.0%. However, only MiKS 3 and MiKS 4 (which had the highest Si concentration) showed higher *A/gs* values, with increases of 102.2% and 31.1%, respectively, when compared to untreated plants.

For chlorophyll *a* fluorescence during the morning period in the maturation of 2024, MiKS 3 and MiKS 4-treated plants showed significantly higher *ΦPSII* values than untreated plants, with increases of 39.8% and 41.7%, respectively. On the other hand, *NPQ* values were significantly higher in MiKS 2-treated plants compared to control (26.0%) and MiKS 3 (27.2%).

Finally, significant differences were observed in water potential during 2024. During veraison, plants treated with all Si and Kl formulations exhibited significantly higher water potential than control plants, with increases of 24.2% for MiKS 2, 42.1% for MiKS 3, and 40.1% for MiKS 4. In the maturation stage, only MiKS 3-treated plants showed a significant increase in water potential compared to untreated plants, with an increase of 17.6%.

**Figure 4:**
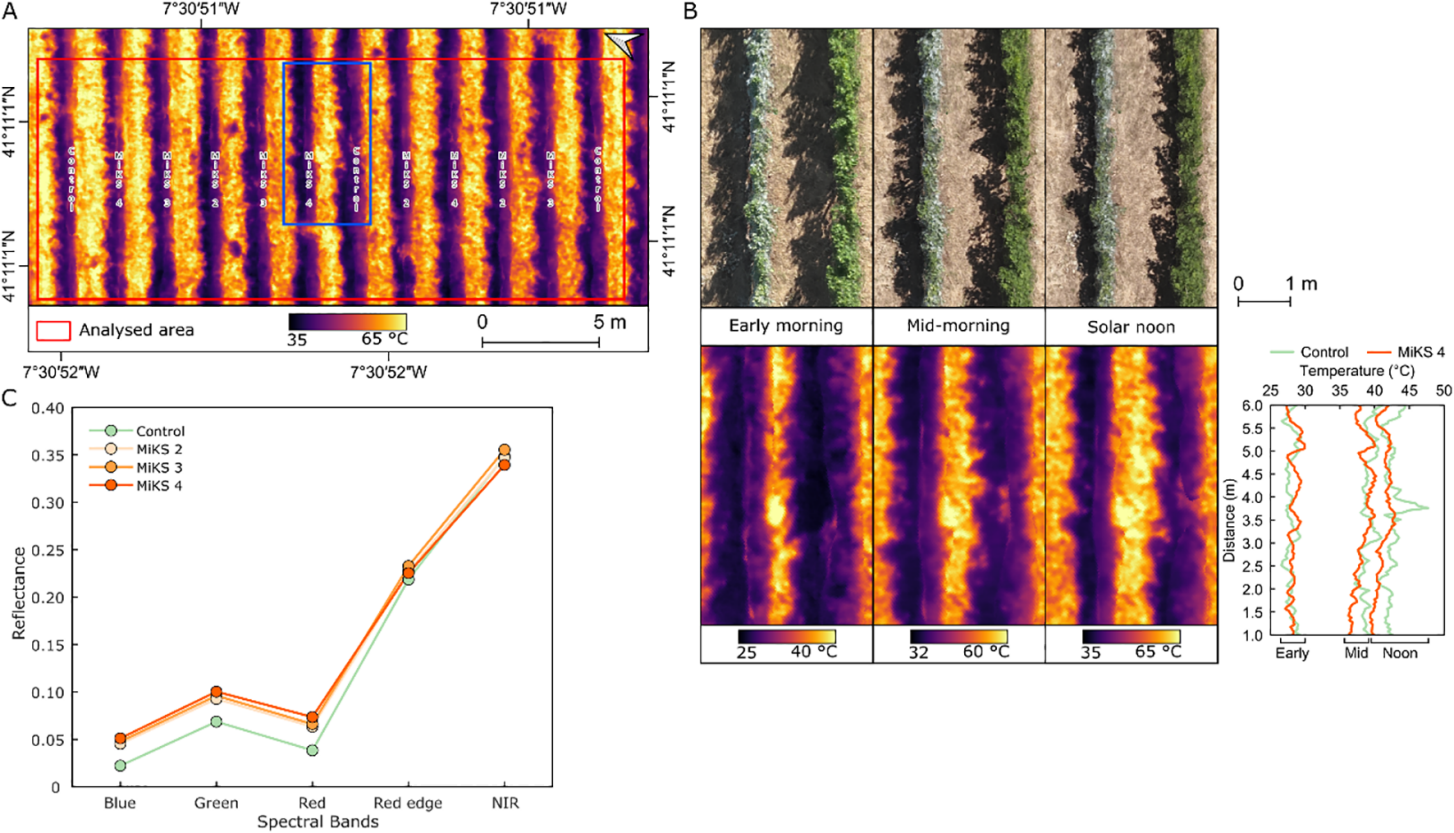
Overview of land surface temperature at solar noon **(A)** during veraison in 2024, in Touriga Franca grapevines subjected to combined application of Kl (2%) and Si (4 to 8%). Detail of two vineyard rows treated with MiKS 4 and control, corresponding to the blue rectangle in A, shown for all three flights, along with the temperature of profile across six-meter transect within the rows **(B)**. Spectral reflectance in the five multispectral bands acquired by the unmanned aerial vehicle **(C)**, n = 56 for control; n = 58 for MiKS 2; n = 59 for MiKS 3; and n = 50 for MiKS 4 (differences in n due to the exclusion of row sections with missing grapevines).

The UAV-based TIR imagery acquired at three time points (morning, mid-morning, and solar noon, presented in **Figure 4A**) during veraison in 2024 showed a variation in grapevine canopy temperature and CWSI among treatments (**Table 3**). Across all flight periods, control plants presented the highest mean canopy temperatures (30.6°C in early morning, 41.0°C in mid-morning, and 44.4°C at solar noon). However, statistical analysis (P-values: 0.82, 0.75, and 0.77, respectively) revealed no significant differences among treatments. In contrast, MiKS-treated plants showed lower temperatures, with MiKS 2 and MiKS 4 recording the lowest early morning values (29.6 °C and 29.7 °C, respectively).

**Table 3:** Grapevine temperature and crop water stress index (CWSI) values (mean ± SD) from thermal infrared data acquired by unmanned aerial vehicle at three different flight periods in veraison and maturation of 2024, in Touriga Franca leaves subjected to combined application of Kl (2%) and Si (4 to 8%).

Treatment	*Temperature (°C)*			*CWSI*		
	*Early morning*	*Mid-morning*	*Solar noon*	*Early morning*	*Mid-morning*	*Solar noon*
**Control**	30.6 ± 0.9	41.0 ± 1.3	44.4 ± 1.2	0.515 ± 0.084	0.753 ± 0.115	0.777 ± 0.128
**MiKS 2 (Kl_2% + Si_4%)**	29.6 ± 0.9	39.8 ± 1.5	43.2 ± 1.5	0.403 ± 0.111	0.652 ± 0.123	0.655 ± 0.133
**MiKS 3 (Kl_2% + Si_6%)**	30.4 ± 0.9	40.6 ± 1.1	43.9 ± 1.1	0.485 ± 0.083	0.713 ± 0.125	0.728 ± 0.147
**MiKS 4 (Kl_2% + Si_8%)**	29.7 ± 0.9	40.4 ± 1.2	44.0 ± 1.1	0.411 ± 0.100	0.701 ± 0.138	0.738 ± 0.143
***P* value**	0.818	0.750	0.769	0.841	0.749	0.771

n = 56 for control; n = 58 for MiKS 2; n = 59 for MiKS 3; and n = 50 for MiKS 4 (differences in n due to the exclusion of row sections with missing grapevines).

In the earlier flight, all MiKS treatments showed lower canopy temperatures compared to the control (30.6°C), as presented in the example in **Figure 4B**. MiKS 2 and MiKS 4 showed the greatest reductions, with a surface temperature decrease of 1.01°C and 0.93°C, respectively. MiKS 3 had a smaller reduction of 0.27°C. At middle-morning flight, the control showed a temperature of 41°C, when comparing to MiKS treatments, MiKS 2 presented the largest decrease (−1.20°C), followed by MiKS 4 (−0.62°C) and MiKS 3 (−0.47°C). At solar noon, all treatments showed lower temperatures than the control (44.4°C), although the differences were generally smaller. MiKS 2 had a 1.16°C reduction, MiKS 3 a 0.47°C reduction, and MiKS 4 a 0.37°C reduction. CWSI values followed a similar trend. The control showed the highest CWSI across all periods, reaching 0.78 at solar noon. MiKS 2 showed the lowest CWSI at all three times (0.40, 0.65, and 0.66), corresponding to reductions of 0.11, 0.10, and 0.12 compared to the control. MiKS 4 also showed CWSI reductions (−0.10 to −0.04), while MiKS 3 values were close to control CWSI values.

The UAV multispectral data (**Figure 4C**) shows that control plants presented a lower reflectance in the visible bands when compared to all MiKS-treated plants. In the blue band, reflectance was 0.02 in the control, while in all MiKS treatments it was 0.05. Similarly, for the green band, the control treatment presented a mean value of 0.07 compared to 0.09–0.10 in the treated groups, and for the red band, the control (0.04) is lower than the 0.06–0.07 observed in the MiKS treatments. In the red edge band, differences were smaller across treatments, with the control at 0.22 and MiKS treatments at around 0.23. NIR reflectance values were also similar with 0.35 for both the control and MiKS 2, 0.36 for MiKS 3, and 0.34 for MiKS 4.

### 3.2 Modulation of leaf anatomy and biochemical defence induced by kaolin–silicon mixtures application

The photosynthetic pigments in leaves treated with different Si + Kl formulations during the veraison and maturation stages of 2023 and 2024 are presented in **Table 4**.

**Table 4:** Photosynthetic pigments (mean±SD): chlorophyll *a*, *b*, *total* and carotenoids, in veraison and maturation of 2023 and 2024, in Touriga Franca leaves subjected to combined application of Kl (2%) and Si (2 to 8%).

Year	Phenological stages	Treatment	Chlorophyll *a* (mg.g^-1^)	Chlorophyll *b* (mg.g^-1^)	Chlorophyll total (mg.g^-1^)	Carotenoids (mg.g^-1^)
**2023**	**Veraison**	**Control**	2.04 ± 0.07 c	1.75 ± 0.03 c	3.79 ± 0.10 c	0.648 ± 0.037 c
		**Miks 1 (Kl_2% + Si_2%)**	1.31 ± 0.06 a	1.37 ± 0.06 a	2.67 ± 0.11 a	0.279 ± 0.017 a
		**Miks 2 (Kl_2% + Si_4%)**	1.79 ± 0.08 b	1.56 ± 0.04 b	3.34 ± 0.12 b	0.501 ± 0.035 b
		**Miks 3 (Kl_2% + Si_6%)**	1.81 ± 0.01 b	1.60 ± 0.02 b	3.40 ± 0.03 b	0.465 ± 0.022 b
		***P* value**	<0.001	<0.001	<0.001	<0.001
	**Maturation**	**Control**	1.59 ± 0.07 b	1.61 ± 0.12 b	3.20 ± 0.19 b	0.387 ± 0.027 bc
		**Miks 1 (Kl_2% + Si_2%)**	1.20 ± 0.05 a	1.35 ± 0.03 a	2.55 ± 0.08 a	0.298 ± 0.024 a
		**Miks 2 (Kl_2% + Si_4%)**	1.57 ± 0.04 b	1.50 ± 0.02 ab	3.07 ± 0.07 b	0.426 ± 0.022 c
		**Miks 3 (Kl_2% + Si_6%)**	1.52 ± 0.01 b	1.52 ± 0.08 ab	3.04 ± 0.08 b	0.343 ± 0.020 ab
		***P* value**	<0.001	0.016	<0.001	<0.001
**2024**	**Veraison**	**Control**	1.59 ± 0.11	1.73 ± 0.22	3.32 ± 0.32	0.485 ± 0.036
		**Miks 2 (Kl_2% + Si_4%)**	1.73 ± 0.07	1.77 ± 0.09	3.50 ± 0.16	0.444 ± 0.022
		**Miks 3 (Kl_2% + Si_6%)**	1.57 ± 0.27	1.67 ± 0.21	3.24 ± 0.48	0.447 ± 0.056
		**Miks 4 (Kl_2% + Si_8%)**	1.71 ± 0.53	1.58 ± 0.12	3.29 ± 0.50	0.525 ± 0.167
		***P* value**	0.301	0.547	0.397	0.544
	**Maturation**	**Control**	1.33 ± 0.18	1.58 ± 0.23	2.91 ± 0.37	0.273 ± 0.110 ab
		**Miks 2 (Kl_2% + Si_4%)**	1.36 ± 0.07	1.55 ± 0.08	2.91 ± 0.15	0.331 ± 0.032 ab
		**Miks 3 (Kl_2% + Si_6%)**	1.23 ± 0.15	1.56 ± 0.25	2.78 ± 0.39	0.240 ± 0.026 a
		**Miks 4 (Kl_2% + Si_8%)**	1.46 ± 0.13	1.62 ± 0.22	3.08 ± 0.35	0.352 ± 0.038 b
		***P* value**	0.067	0.928	0.499	0.018

Different letters represent significant differences between the treatments at the same time of day and within the same phenological stage. The absence of letters indicates that there are no significant differences between treatments. n = 9.

**Figure 5:**
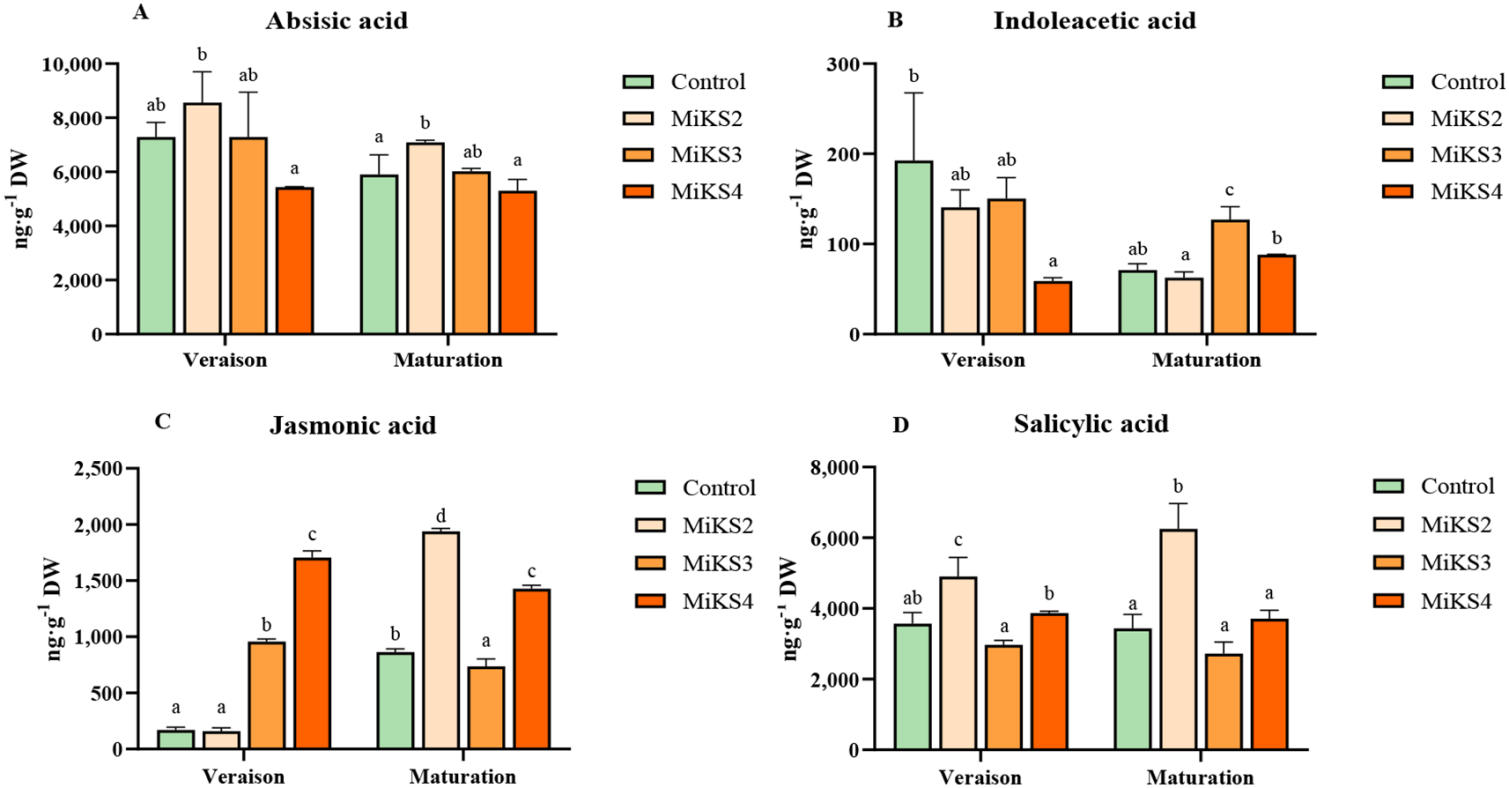
Hormonal profile (mean ± SD): **(A)** abscisic acid, **(B)** indoleacetic acid, **(C)** jasmonic acid, and **(D)** salicylic acid, in veraison and maturation of 2023 and 2024, in Touriga Franca leaves subjected to combined application of Kl (2%) and Si (2 to 8%). Different letters represent significant differences between the treatments within the same phenological stage. The absence of letters indicates that there are no significant differences between treatments. n = 9.

Additionally, **Table 5** displays the results for soluble sugars, soluble proteins, and secondary metabolites (total phenols, total flavonoids, and *ortho*-diphenols) analysed in the same leaves and timepoints. Meanwhile, histological parameters of the leaves were also analysed, and the results are presented in **Table 6**. Regarding the hormonal profile, this was analysed only during the veraison and maturation stages of 2024, and the results are shown in **Figure 5**.

**Table 5:** Primary and secondary metabolites (mean±SD): soluble sugars, soluble protein, total phenols, flavonoids, and *ortho*-diphenols, in veraison and maturation of 2023 and 2024, in Touriga Franca leaves subjected to combined application of Kl (2%) and Si (2 to 8%).

Year	Phenological stages	Treatment	Soluble sugars (mg.g^-1^)	Soluble protein (mg.g^-1^)	Total phenols (mg.g^-1^)	Total flavonoids (mg.g^-1^)	*Ortho*-diphenols (mg.g^-1^)
**2023**	**Veraison**	**Control**	31.8 ± 1.0 b	1.47 ± 0.10	17.5 ± 0.5 a	5.53 ± 0.08 a	32.4 ± 0.4 a
		**Miks 1 (Kl_2% + Si_2%)**	24.9 ± 2.3 a	1.48 ± 0.16	18.9 ± 1.1 ab	6.68 ± 0.29 b	36.7 ± 0.6 b
		**Miks 2 (Kl_2% + Si_4%)**	34.2 ± 2.2 b	1.52 ± 0.14	20.1 ± 1.1 b	7.82 ± 0.80 c	44.4 ± 1.3 c
		**Miks 3 (Kl_2% + Si_6%)**	33.7 ± 2.0 b	1.42 ± 0.11	19.4 ± 1.0 ab	6.84 ± 0.09 bc	44.3 ± 0.8 c
		***P* value**	<0.001	0.632	0.048	0.001	<0.001
	**Maturation**	**Control**	41.5 ± 2.6 c	1.58 ± 0.11	17.5 ± 0.6 ab	5.36 ± 0.09 a	37.0 ± 1.6 b
		**Miks 1 (Kl_2% + Si_2%)**	33.7 ± 1.7 a	1.60 ± 0.14	18.9 ± 0.8 bc	6.22 ± 0.18 a	37.8 ± 1.3 b
		**Miks 2 (Kl_2% + Si_4%)**	38.3 ± 1.2 b	1.68 ± 0.10	16.7 ± 0.2 a	5.56 ± 0.01 a	32.4 ± 1.2 a
		**Miks 3 (Kl_2% + Si_6%)**	41.4 ± 1.9 c	1.72 ± 0.13	20.6 ± 0.9 c	7.29 ± 0.71 b	41.8 ± 0.9 c
		***P* value**	<0.001	0.199	<0.001	<0.001	<0.001
**2024**	**Veraison**	**Control**	32.8 ± 1.3 a	1.51 ± 0.10 ab	16.6 ± 1.8	4.43 ± 0.25 a	31.0 ± 1.9 ab
		**Miks 2 (Kl_2% + Si_4%)**	42.9 ± 1.8 c	1.74 ± 0.14 c	15.9 ± 0.9	4.48 ± 0.17 ab	31.2 ± 1.0 ab
		**Miks 3 (Kl_2% + Si_6%)**	43.9 ± 1.2 c	1.45 ± 0.08 a	17.6 ± 1.7	5.18 ± 0.18 c	33.1 ± 1.6 b
		**Miks 4 (Kl_2% + Si_8%)**	38.2 ± 1.4 b	1.63 ± 0.09 bc	18.0 ± 1.9	4.72 ± 0.19 b	30.3 ± 1.1 a
		***P* value**	<0.001	<0.001	0.150	<0.001	0.019
	**Maturation**	**Control**	35.6 ± 1.2 c	1.62 ± 0.15 a	16.6 ± 1.9 a	3.51 ± 0.42 a	26.5 ± 1.2 a
		**Miks 2 (Kl_2% + Si_4%)**	24.6 ± 2.1 a	1.27 ± 0.11 a	16.0 ± 1.9 a	3.73 ± 0.25 ab	25.2 ± 1.1 a
		**Miks 3 (Kl_2% + Si_6%)**	36.6 ± 1.7 c	1.61 ± 0.05 a	16.9 ± 2.1 a	4.22 ± 0.49 b	29.2 ± 1.8 b
		**Miks 4 (Kl_2% + Si_8%)**	28.9 ± 1.3 b	2.42 ± 0.40 b	25.4 ± 0.8 b	5.99 ± 0.75 c	34.8 ± 1.5 c
		***P* value**	<0.001	<0.001	<0.001	<0.001	<0.001

Different letters represent significant differences between the treatments at the same time of day and within the same phenological stage. The absence of letters indicates that there are no significant differences between treatments. n = 9.

**Table 6:** Histological parameters (mean ± SD): total leaf thickness, upper and lower cuticle, upper and lower epidermis, palisade and spongy mesophyll cells, and mesophyll thickness, in veraison and maturation of 2023 and 2024, in Touriga Franca leaves subjected to combined application of Kl (2%) and Si (2 to 8%).

Year	Phenological stages	Treatment	Total leaf thickness (µm)	Upper cuticle (µm)	Lower cuticle (µm)	Upper epidermis (µm)	Lower epidermis (µm)	Palisade mesophyll cells (µm)	Spongy mesophyll cells (µm)	Mesophyll thickness (µm)
2023	**Veraison**	**Control**	122.0 ± 12.9	2.81 ± 0.39 a	2.40 ± 0.12 a	11.7 ± 2.0	13.3 ± 2.6	43.2 ± 5.4	48.7 ± 9.9	91.9 ± 13.5
		**Miks 1 (Kl_2% + Si_2%)**	125.4 ± 9.3	3.14 ± 0.29 ab	2.43 ± 0.19 a	12.8 ± 1.6	14.2 ± 4.2	40.1 ± 4.1	52.7 ± 9.7	92.8 ± 9.2
		**Miks 2 (Kl_2% + Si_4%)**	134.9 ± 16.9	3.32 ± 0.21 b	2.87 ± 0.27 b	12.0 ± 1.7	16.6 ± 4.5	46.7 ± 8.4	53.4 ± 9.1	100.1 ± 16.4
		**Miks 3 (Kl_2% + Si_6%)**	139.7 ± 15.6	3.38 ± 0.16 b	2.93 ± 0.27 b	12.0 ± 2.9	14.5 ± 3.6	43.9 ± 3.6	63.1 ± 8.4	107.0 ± 11.6
		***P* value**	0.135	0.008	<0.001	0.780	0.482	0.275	0.080	0.183
	**Maturation**	**Control**	125.5 ± 18.2	2.81 ± 0.23 a	2.40 ± 0.24 a	14.9 ± 3.3	12.7 ± 5.4	40.8 ± 7.1	51.8 ± 11.0	92.6 ± 12.9
		**Miks 1 (Kl_2% + Si_2%)**	129.0 ± 8.4	3.13 ± 0.18 b	2.75 ± 0.27 ab	13.3 ± 2.6	14.1 ± 2.5	41.8 ± 4.3	54.0 ± 8.5	95.8 ± 7.5
		**Miks 2 (Kl_2% + Si_4%)**	130.7 ± 15.6	3.59 ± 0.22 c	2.93 ± 0.28 b	14.3 ± 2.2	14.5 ± 3.9	46.2 ± 9.3	49.3 ± 7.0	95.5 ± 12.7
		**Miks 3 (Kl_2% + Si_6%)**	137.5 ± 20.8	3.72 ± 0.15 c	2.92 ± 0.24 b	14.8 ± 2.5	16.2 ± 2.8	45.4 ± 6.9	54.4 ± 14.9	99.9 ± 21.4
		***P* value**	0.644	<0.001	0.007	0.716	0.487	0.488	0.831	0.854
2024	**Veraison**	**Control**	173.6 ± 10.9 ab	3.47 ± 0.45 a	3.01 ± 0.46 a	15.8 ± 3.0 b	14.2 ± 3.8	54.7 ± 6.0 a	82.3 ± 11.0	137.0 ± 9.0 a
		**Miks 2 (Kl_2% + Si_4%)**	188.2 ± 22.5 b	4.25 ± 0.61 b	3.72 ± 0.56 b	12.4 ± 2.6 a	13.4 ± 2.6	66.7 ± 16.4 b	87.8 ± 15.8	154.4 ± 19.7 b
		**Miks 3 (Kl_2% + Si_6%)**	178.6 ± 15.3 ab	4.59 ± 0.60 b	3.61 ± 0.44 b	12.9 ± 2.8 ab	15.0 ± 3.3	49.4 ± 3.9 a	93.1 ± 14.0	142.5 ± 14.6 ab
		**Miks 4 (Kl_2% + Si_8%)**	169.1 ± 12.3 a	4.36 ± 0.55 b	3.61 ± 0.67 b	14.9 ± 3.7 ab	13.3 ± 2.3	48.7 ± 6.6 a	84.2 ± 6.6	133.0 ± 11.9 a
		***P* value**	0.033	<0.001	0.010	0.023	0.480	<0.001	0.168	0.004
	**Maturation**	**Control**	163.4 ± 13.9	2.98 ± 0.33 a	2.49 ± 0.42 a	16.0 ± 3.5 b	14.7 ± 4.4	49.1 ± 5.9 a	78.1 ± 11.7	127.3 ± 13.9 a
		**Miks 2 (Kl_2% + Si_4%)**	177.9 ± 18.4	4.21 ± 0.59 b	3.48 ± 0.45 b	13.8 ± 2.6 ab	12.2 ± 2.5	55.9 ± 9.0 a	88.3 ± 16.6	144.2 ± 18.9 ab
		**Miks 3 (Kl_2% + Si_6%)**	181.1 ± 22.3	4.50 ± 0.50 b	3.78 ± 0.63 b	11.8 ± 2.5 a	14.1 ± 3.9	65.8 ± 9.4 b	81.1 ± 15.4	146.9 ± 18.9 b
		**Miks 4 (Kl_2% + Si_8%)**	177.8 ± 15.3	4.52 ± 0.40 b	3.38 ± 0.38 b	13.1 ± 2.1 ab	13.1 ± 2.2	55.2 ± 9.1 a	88.5 ± 14.6	143.7 ± 14.9 ab
		***P* value**	0.083	<0.001	<0.001	0.005	0.304	<0.001	0.226	0.026

Different letters represent significant differences between the treatments at the same time of day and within the same phenological stage. The absence of letters indicates that there are no significant differences between treatments.

n = 9.

During the veraison stage in 2023, significant differences were observed across several physiological and biochemical parameters in grapevine leaves. For photosynthetic pigments (**Table 4**), untreated plants showed the highest levels of chlorophyll *a*, chlorophyll *b*, total chlorophyll, and carotenoids, while MiKS 1-treated plants exhibited the lowest. MiKS 2 and MiKS 3 treatments resulted in intermediate values, being significantly lower than the control but higher than MiKS 1. Regarding sugar content, untreated, MiKS 2-, and MiKS 3-treated plants had significantly higher levels than MiKS 1-treated plants, with increases of 27.7%, 37.3%, and 35.3%, respectively. MiKS 2-treated plants also showed a significant increase in both total phenol content (14.5%) and flavonoid levels (41.4%) in comparison to the control. All treatments significantly enhanced *ortho*-diphenol content compared to control plants, with increases of 13.3% (MiKS 1), 37.1% (MiKS 2), and 36.7% (MiKS 3).

At maturation in 2023, several parameters continued to show significant variation among treatments. Photosynthetic pigment levels remained the lowest in MiKS 1-treated plants. The control plants maintained the highest levels of all pigments except for carotenoids. MiKS 2 and MiKS 3 treatments increased chlorophyll *a* and total chlorophyll levels, with MiKS 2 also leading to a higher carotenoid content. Sugar content (**Table 5**) followed the same pattern observed in veraison: MiKS 1-treated plants showed the lowest values, while control, MiKS 2, and MiKS 3 treatments resulted in increases of 23.1%, 13.6%, and 22.8%, respectively. MiKS 3-treated plants displayed the highest levels of total phenols, flavonoids, and *ortho*-diphenols. For this treatment, total phenol content increased by 23.2% and 17.8% when compared to MiKS 2 and control, respectively. Flavonoid content also rose by 36.0%, 17.2%, and 31.1% relative to the control, MiKS 1, and MiKS 2, respectively. Additionally, *ortho*-diphenol levels in MiKS 3-treated plants increased by 29.1%, 10.6%, and 13.0% compared to MiKS 2, MiKS 1, and control, respectively. In the same year, for the histological parameters, significant differences were observed in the upper cuticle and lower cuticle at both phenological stages, with MiKS 2- and MiKS 3-treated plants showing the highest values.

During the veraison stage of 2024, significant differences were found in sugar, protein, flavonoids, *ortho*-diphenols, and phytohormones. Sugar content was significantly higher in all treated plants compared to the control, with increases of 30.8% (MiKS 2), 33.8% (MiKS 3), and 16.5% (MiKS 4). Protein levels were significantly higher in MiKS 2-treated plants than in MiKS 3, with a 20.0% increase. Flavonoid content was highest in MiKS 3-treated plants, exhibiting a 16.9% increase when compared to the control. These plants also showed the highest *ortho*-diphenol content, with a 9.26% increase relative to MiKS 4-treated plants. Phytohormone analysis (**Figure 5**) revealed that, at veraison, MiKS 2-treated plants had the highest ABA content, showing a 57.4% increase compared to MiKS 4. Furthermore, MiKS 4-treated plants also had the lowest IAA levels, 69.4% lower than the control. JA content was markedly increased in MiKS 3 and MiKS 4 (463.7% and 904.0% increases, respectively, over the control). For SA, MiKS 2 and MiKS 4 treatments showed increases of 65.0% and 30.2%, respectively, compared to MiKS 3, with MiKS 2 also surpassing the control by 37.5%. In this phenological stage, both the upper and lower cuticles exhibited the same pattern as in 2023, with all treated plants showing a significant increase in these parameters compared to the untreated ones (**Table 6**). Furthermore, MiKS 2-treated plants demonstrated significantly higher palisade mesophyll cell density, total leaf thickness, and mesophyll thickness, while MiKS 4-treated plants consistently displayed the lowest values. Notably, control plants had a significantly thicker upper epidermis than MiKS 2-treated plants, with a 27.4% increase.

At the maturation stage in 2024, significant differences were observed in pigment composition, sugar, protein, phenolic compounds, phytohormones, and histological traits. Carotenoid content was significantly higher in MiKS 4-treated plants than in MiKS 3, with a 46.7% increase (**Table 4**). Sugar levels were highest in control, MiKS 3-, and MiKS 4-treated plants, all significantly surpassing MiKS 2 with increases of 44.7%, 48.8%, and 17.5%, respectively (**Table 5**). MiKS 4-treated plants exhibited the highest contents of protein, phenols, flavonoids, and *ortho*-diphenols. Protein content was particularly elevated, with increases of 49.4%, 90.6%, and 50.3% compared to the control, MiKS 2, and MiKS 3, respectively. Similarly, total phenol content was highest in MiKS 4-treated plants, surpassing the control, MiKS 2, and MiKS 3 by 52.7%, 58.9%, and 50.2%, respectively. Flavonoid levels followed a similar pattern, with MiKS 4 showing increases of 70.7%, 60.6%, and 41.9% compared to the control, MiKS 2, and MiKS 3. MiKS 3-treated plants also exhibited significantly higher flavonoid content than the control, with a 20.2% increase. Moreover, MiKS 4-treated plants had the highest *ortho*-diphenol content, exceeding the control, MiKS 2, and MiKS 3 by 31.3%, 38.0%, and 19.2%, respectively. MiKS 3 also had higher content of *ortho*-diphenol than the control (10.2%) and MiKS 2 (15.8%). Phytohormonal analysis (**Figure 5**) revealed that, at maturation, MiKS 2 maintained the highest ABA levels, with increases of 19.98% and 33.58% compared to control and MiKS 4, respectively. MiKS 3 exhibited the highest IAA content, surpassing the control by 78.8%, MiKS 2 by 102.9%, and MiKS 4 by 44.1%. Relative to the control, JA levels were significantly increased in MiKS 2 (124.2%) and MiKS 4 (65.2%), while MiKS 3 showed a 14.8% decrease. SA content was highest in MiKS 2-treated plants, with increases of 82.0%, 129.7%, and 68.5% compared to control, MiKS 3, and MiKS 4, respectively. Similar to the veraison stage, during maturation, both the upper and lower cuticles maintained the same pattern, with all treated plants showing a significant increase in these parameters compared to untreated ones (**Table 6**). Moreover, MiKS 3-treated plants exhibited significantly higher palisade mesophyll cell density and, as a result, greater mesophyll thickness, particularly when compared to control plants, with increases of 34.0% and 15.4%, respectively. On the other hand, MiKS 3-treated plants simultaneously displayed the thinnest upper epidermis compared to control plants, with a 26.3% reduction.

## 4 Discussion

The combination of treatments with Si and Kl demonstrated a significant impact on the ecophysiological and biochemical performance of grapevines, particularly under summer stress conditions. The analysis of these parameters revealed that treatments not only optimized water-use efficiency and photosynthetic performance but also enhanced the plant’s biochemical defences, improving overall resilience.

### 4.1 Physiological performance of grapevines under summer stress pulverized by Kaolin–silicon mixtures

The application of Si and Kl significantly improved gas exchange parameters, particularly under abiotic stress conditions. The contrasting *gs* responses observed between 2023 and 2024 can be largely explained by the markedly different climatic conditions registered during both growing seasons. As shown in **Figure 1**, 2023 was characterized by extremely low precipitation during the vegetative and ripening phases, coupled with consistently high temperatures, particularly during the summer months. These conditions imposed significant water stress on the vines, strongly limiting stomatal conductance. Under such circumstances, MiKS 2 exhibited the highest *gs* values. This formulation may have permitted a more balanced stomatal behaviour. The moderate antitranspirant effect provided by MiKS 2 possibly reduced excessive water loss while still allowing sufficient stomatal opening for gas exchange under severe drought, thus optimizing physiological performance under high vapor pressure deficit conditions. On the other hand, in 2024, precipitation was more frequent throughout the vegetative cycle, with rainfall episodes occurring between April and July. The increased soil water availability likely alleviated water stress, allowing treatments MiKS 3 and MiKS 4 to exert their beneficial effects by mitigating heat load and photoinhibition through increased leaf reflectance. The increase in stomatal conductance led to a higher *A* due to greater CO_2_ uptake, which consequently also resulted in an increased *E* (**Table 1**). These findings confirm the role of Si in maintaining stomatal functionality during periods of high temperatures, potentially by enhancing water uptake and transport (Laane, 2018), which positively influences photosynthesis (Khan et al., 2017; Tripathi et al., 2017). Additionally, Si role in enhancing the synthesis of aquaporins might also support its role in improving water uptake, which would further enhance gas exchange under heat stress (Wang et al., 2021). Moreover, Kl may have contributed to this effect by improving photosynthetic activity under drought and heat stress, as supported by Cataldo et al. (2022). Pilon et al. (2013) reported that an increase in *A* in Si-treated plants was associated with higher *gs* and increased concentrations of chlorophyll *a* and carotenoids. Interestingly, in this study, grapevines treated with the highest Si concentration, particularly MiKS 4, exhibited increased *gs* and higher carotenoid levels during maturation in 2024 (**Table 3**). Although chlorophyll *a* levels also increased, the differences were not statistically significant. Higher stomatal conductance allows for greater stomatal opening, enhancing CO_2_ assimilation and increasing photosynthesis, as CO_2_ is essential for glucose production in the Calvin cycle. However, this increase also leads to higher water loss through *E*. While increased *gs* improves CO_2_ uptake, plants must regulate water loss to maintain water-use efficiency (Giorgi et al., 2019). Thus, the formulation MiKS 4 played a role in keeping stomatal function active, increasing photosynthesis and transpiration and enabling greater gas exchange. The trade-off between photosynthesis and water loss is a complex adaptive mechanism that may be modulated by the enhanced efficiency of the cuticle, as shown in the histological analysis (**Table 6**).

Although statistically significant increases in *A/gs* ratio were observed specifically at solar noon during both veraison and maturation in 2024, these results suggest that, under certain phenological stages and peak irradiance periods, combined application with Si and Kl may contribute to a more efficient water use. Indeed, several studies have reported improvements in water use efficiency across a variety of crops following foliar Si application (Ma et al., 2004; Gunes et al., 2008; Ming et al., 2012; Ali et al., 2013; Amin et al., 2014; Ahmed et al., 2014; Kurdali et al., 2013; Dehghanipoodeh et al., 2018). This enhancement is often attributed to increased leaf and epidermal tissue thickness due to the deposition of a silica double layer (Gong et al., 2003). The deposition of Si not only strengthens the cuticular layer but also may contribute to improved water retention in leaf tissues, directly supporting the plants’ ability to withstand water stress. Similarly, Kl application has also been reported to improve water use efficiency in vineyards by reducing water loss through the lowering of leaf temperature (Glenn et al., 2010; Jifon and Syvertsen, 2003), although variable responses have been observed in other studies (Segura-Monroy et al., 2015).

Predawn leaf water potential is widely recognized as a simple yet reliable physiological indicator for assessing grapevine water status (Levin, 2019; Tuccio et al., 2019). The absence of significant differences in predawn water potential among treatments in 2023, despite the severe drought conditions, suggests that under extreme water deficit, the capacity of the applied treatments to improve vine water status may be limited. In such conditions, soil water availability may be so low that any potential benefit from treatments like Si and Kl is overridden by the prevailing water scarcity. On the other hand, the increases in predawn water potential in 2024 (**Figure 3**) for treated plants suggest, in agreement with *A/gs*, that these treatments helped the plants maintain a better water status, which is critical for survival during drought periods. In fact, in the present study, the water potential exhibited a similar trend to *gs*, *A*, and *E*, as all these parameters are closely regulated by stomatal behaviour, which is influenced by the plant’s water status. When water availability is high, stomata remain open, facilitating gas exchange and optimizing both photosynthesis and transpiration (Zhu et al., 2023). This suggests that the treatments did not only preserve stomatal function but also improved the plant’s internal hydraulic conductance, further supporting better water retention and efficiency. An increase in water potential as a result of Si application has been also reported in studies across various crops (Gong and Chen, 2012; Ming et al., 2012; Hattori et al., 2007). According to Rizwan et al. (2015), Si accumulates in the leaf epidermis forming a physical barrier that reduces water loss. Similarly, the improvement in plant water status through Kl application under stress conditions, as reported in several studies (Glenn et al., 2010; Denaxa et al., 2012; Boari et al., 2015; Nanos, 2015; AbdAllah, 2017; Dinis et al., 2018a; Brito et al., 2018, 2019), is mainly attributed to the formation of a reflective particle film on the leaf surface, which lowers leaf temperature and helps maintain higher leaf water potential under stress.

According to Ojeda et al. (2001), the level of water stress experienced by a plant can be assessed based on baseline water potential values. In this study, the grapevines were classified as experiencing moderate water stress at veraison in 2023. By veraison in 2024, water potential values had become less negative across all treatments, with treated plants even reaching a state of hydric comfort (0 to -0.3 MPa). Although similar trends were observed in the control group, the significant increases observed in treated plants suggest an improved capacity for water retention, helping to mitigate the negative effects of water stress and sustain physiological functions during critical phenological stages. However, as the maturation stage progressed, water potential values dropped below -0.6 MPa in both years, indicating that the plants were subjected to moderate water stress at this stage (Ojeda et al., 2001). Nevertheless, the treatments with MiKS 2 and MiKS 3 showed slightly higher values than -0.6 MPa, in maturation 2024. Prichard et al. (2004) have suggested that moderate water stress in red wine grape varieties was beneficial for improving must quality, as it stimulates the synthesis of polyphenols, aromatic compounds and other beneficial compounds, particularly during veraison. This controlled stress encourages the development of complex flavours and the accumulation of compounds that contribute to the wine’s colour, tannin structure, and aromatic profile. However, excessive water availability can disrupt this beneficial stress response. By increasing water supply, the mobilization of photoassimilates to the berries is reduced, which may hinder the concentration of key compounds and negatively affect fruit quality, potentially leading to wines with less intensity and complexity (Magalhães, 2015).

In 2023, extreme drought and heat likely triggered a protective downregulation of PSII efficiency in grapevines, masking potential treatment effects. On the contrary, during veraison and maturation in 2024, plants treated with MiKS 3 and MiKS 4 demonstrated higher *ΦPSII* values (**Table 2**), which can be related to the protection of the photosynthetic apparatus (Qin et al., 2016). Indeed, this increase suggests that treatments applied in the current study improved photosynthetic efficiency, allowing the plants to maximize their energy production despite stressful environmental conditions. These treatments showed reduced susceptibility to photoinhibition, likely due to the enhanced efficiency of photosystem II and, in some cases, a greater capacity for efficient photochemical quenching (Dinis et al., 2018a). This improvement is probably linked to increased sucrose concentration in the leaves (as observed in the present study), enhanced sucrose transport, and greater phloem loading ability (Conde et al., 2016, 2018). Furthermore, this enhanced photochemical efficiency could also be partly attributed to the hormonal shifts induced by the treatments, particularly the reduction in ABA and the increase in IAA, which together support a more resilient photosynthetic system under stress (Li et al., 2019; Muller et al., 2021). Recent studies conducted on table grapes and wheat in dry and arid climates have confirmed the beneficial effects on fluorescence parameters after the application of Si (Maghsoudi et al., 2015; Nascimento et al., 2022). Indeed, both studies revealed that the plants suffered less damage to the photosynthetic apparatus, improving their performance when subjected to water and heat stress conditions. Similarly, Kl application can positively influence *PSII* functionality by acting as a reflective barrier, reducing excess light absorption and lowering leaf temperature (Azuara et al., 2023). Kl helps prevent the overexcitation of chlorophyll molecules by reflecting a portion of the incoming solar radiation, thus reducing the risk of photoinhibition and oxidative stress in the photosynthetic machinery (Rosati et al., 2006). The general increase in *NPQ* observed in plants treated with Si + Kl, especially during veraison in 2024, suggests an improved capacity to dissipate the excess of light energy as heat through non-photochemical quenching mechanisms. This process is crucial under stress conditions, as it allows plants to safely release the surplus energy that cannot be used for photochemistry, thereby preventing the overexcitation of chlorophyll molecules and the generation of reactive oxygen species (ROS) (Murakami et al., 2024).

UAV-based TIR data (**Table 3**) demonstrated that foliar application of Kl and Si, contributed to reduced canopy temperature and showed lower CWSI values during veraison in 2024, particularly in MiKS 2-treated plants. Although the differences were not statistically significant, the reduction in canopy temperature and CWSI values across treated grapevines suggests an improved capacity to buffer physiological stress under high thermal load. In the early morning, MiKS 2 and MiKS 4 showed the greatest temperature reductions compared to the control, indicating an improved canopy cooling at the start of the day. Mid-morning measurements revealed that foliar treatments continued to moderate canopy warming as solar radiation increased. At solar noon (**Figure 4A**), despite peak thermal stress, all treatments maintained slightly lower values than the control, with MiKS 2-treated plants showing the greatest difference and the most consistent effects across temperature and CWSI, demonstrating suitability to improve grapevine resilience under climate-related stress conditions. These observations point to a cumulative mitigation effect, whereby foliar applications reduced the impact of thermal stress during the critical periods (Rodrigues et al., 2025). Multispectral reflectance data (**Figure 4C**) further support the influence of Kl and Si treatments on canopy physiology and structure (Okhrimenko et al., 2019). Treated grapevines showed higher reflectance in the visible bands (blue, green, and red) compared to the control, which may be indicative of changes in pigment concentration or leaf surface characteristics, as observed in this work (**Tables 4**, **6**) (Cabello-Pasini and Macías-Carranza, 2011; Albetis et al., 2018). Among the treatments, MiKS 2-treated plants presented slightly lower green and red reflectance compared to MiKS 3 and MiKS 4, which may reflect a dose-dependent effect of Si on pigment-related traits. In the red edge and NIR bands, all treatments showed similar reflectance values. Overall, the integration of UAV-based TIR and multispectral data demonstrated that Kl and Si foliar applications modulate both canopy temperature dynamics and optical properties, serving as a complementary approach to conventional measurements (Pádua et al., 2022). These remotely sensed indicators align with higher *gs*, *A*, and predawn water potential observed in the treated grapevines (**Table 1**, **Figure 3**). UAV-based data thus provide a scalable, non-destructive means to assess vine physiological status and treatment efficacy.

### 4.2 Mixture of kaolin–silicon application modulates leaf tissues thickness and the biochemical defence

The biochemical parameters, including photosynthetic pigments, soluble sugars, soluble proteins, and secondary metabolites, showed a clear beneficial response to Si and Kl treatments.

In 2023, although MiKS 2 and MiKS 3-treated plants had significantly higher chlorophyll content compared to MiKS 1-treated plants, the untreated control plants exhibited even greater chlorophyll levels (**Table 4**). This response in untreated plants might also indicate an overcompensation for the stress, which could result in lower overall efficiency in photosynthesis due to the lack of additional protective mechanisms like those provided by Si and Kl treatments (Anjum et al., 2011; Ashraf and Harris, 2013).

In 2024, no significant differences were observed among the different treatments. Thus, the enhanced photosynthetic performance observed is likely attributable to other factors than pigment concentration, as the treatments did not significantly increase photosynthetic pigments, such as chlorophylls. Nevertheless, the improvement in photosynthetic efficiency can be attributed to the broader physiological benefits conferred by Si and Kl applications, which contributed to optimizing the photosynthetic process under stress conditions (Ma and Yamaji, 2015; Coskun et al., 2018; Dinis et al., 2018a). In fact, in the work carried out by Zuccarini (2008), Si application led to an increase in photosynthetic rate without significant changes in chlorophyll content, suggesting that Si may improve photosynthetic efficiency by maintaining cell membrane integrity and regulating stomatal conductance rather than by directly enhancing pigment levels. Regarding carotenoids, MiKS 2 exhibited higher levels during maturation in 2023, whereas at veraison, its levels were intermediate, falling below those of the control. However, by maturation in 2024, MiKS 4 had the highest carotenoid content. An increase in carotenoids benefits plants by protecting them from excess light, dissipating energy, and preventing damage to *PSII* (Pitawala et al., 2024). Additionally, their antioxidant properties help reduce oxidative stress caused by drought, heat, and intense radiation. Carotenoids also stabilize photosynthesis by improving light absorption and energy efficiency. In this way, plants with higher carotenoid levels are more resilient and productive under adverse conditions. In fact, a lower total carotenoid content is associated with a reduced availability of photoprotective pigments within the cells, limiting the plant’s ability to effectively dissipate excess light energy and neutralize reactive oxygen species. As a result, plants with insufficient carotenoid levels are more susceptible to photodamage when exposed to environmental stresses, such as high light intensity, drought, or heat, ultimately compromising the integrity and efficiency of the photosynthetic apparatus (Pitawala et al., 2024).

Regarding to leaf biochemical attributes (**Table 5**), soluble sugars in MiKS 3-treated plants were significantly higher compared to untreated plants during both veraison and maturation stages, which indicates an enhanced ability to accumulate carbohydrates as a protective mechanism against summer stress. These sugars likely serve as energy reserves, helping the plant to manage stress. Similar results, with an increase of soluble sugar content, were observed in several studies after the Si application (Ding et al., 2007; Trejo-Téllez et al., 2020; Camargo et al., 2023) and Kl application (Bernardo et al., 2021), which can be attributed to improved photosynthetic efficiency, better water use efficiency, and enhanced protection against abiotic stresses, which may have led to higher carbohydrate production and accumulation in the plants. In the present study, the increase in soluble sugars was accompanied by higher levels of soluble proteins, particularly in MiKS 4-treated plants in 2024. Increases in soluble proteins could be essential for stress responses, aiding in cellular maintenance and enzymatic activities that mitigate damage caused by environmental stressors. In fact, this accumulation of soluble protein has been previously observed after Si (Ding et al., 2007; Trejo-Téllez et al., 2020; Dinis et al., 2024) and Kl applications (Brito et al., 2018; Dinis et al., 2018a; Dinis et al., 2020).

Regarding secondary metabolites (**Table 5**), significant increases in phenols, flavonoids, and *ortho*-diphenols were generally observed in treated plants across both phenological stages and years, except for total phenols at veraison 2024 (no significant differences) and *ortho*-diphenols at maturation in 2023 (where only MiKS 3 showed significantly higher levels compared to the control). These compounds are known for their antioxidant properties, playing a crucial role in protecting plants against oxidative damage induced by stress. The observed biochemical enhancements can be attributed to the synergistic effects of Si and Kl applications. Indeed, these compounds are widely recognized for boosting plant resistance to stress and promoting the synthesis of secondary metabolites (Jiang et al., 2022; Dinis et al., 2024; Paskovic et al., 2024).

The observed enhancement of antioxidant defences, photosynthetic capacity, and osmotic regulation in treated plants may be closely linked to hormonal modulation (**Figure 5**), particularly a slight reduction in abscisic acid (ABA) levels in MiKS 4 and an increase in MiKS 2-treated plants, and an increase in indole-3-acetic acid (IAA) in MiKS 3- and MiKS 4-treated plants. These hormonal adjustments likely contributed to greater growth plasticity and improved adaptability under stress conditions (Dinis et al., 2018b). Fast hormonal responses are essential for plant acclimation/adaptation at both the physiological and biochemical levels (Jesus et al., 2015). In the context of summer stress, which is typical in the Douro Region, stomatal closure represents a short-term adaptive response. This process is tightly regulated by a complex network of signalling pathways, including hormonal regulation (Moutinho-Pereira et al., 2004; Zhang et al., 2006; Wani et al., 2016). Specifically, ABA regulates stomatal opening and closure, as well as leaf growth. It is synthesized in the mature chloroplasts of leaves (Pei et al., 2000) and/or in drying roots, then transported via xylem to the leaves (Zhang et al., 2006; Correia et al., 2014). In the present work, plants treated with MiKS 4 exhibited the lowest ABA levels, reflecting the better water status and higher stomatal conductance of these plants (**Figure 5**). This observation is aligned with findings of other authors, who reported that Kl application can reduce ABA biosynthesis in grapevines (Dinis et al., 2018b; Frioni et al., 2020). Furthermore, a reduction in ABA levels was also observed following Si application (Helaly et al., 2017; Hosseini et al., 2017). Conversely, the increase in ABA levels observed in MiKS 2-treated plants may suggest a lower effectiveness in mitigating water stress, as ABA accumulation generally reflects drought perception and stomatal closure (Chaves et al., 2009).

IAA, the main auxin in plants, is a key hormone involved in regulating cell growth, development, and root patterning (Dinis et al., 2018b; Zhao, 2010). Thus, the increased IAA levels observed in MiKS 3and MiKS 4-treated plants (**Figure 5**) indicate enhanced growth and cell division, supporting overall plant development and stress resistance. This overproduction of IAA following Kl application suggests improved acclimation to environmental stresses, according to previous findings (Ke et al., 2015; Wani et al., 2016). Similarly, several studies have reported an increase in IAA content after Si application (Wani et al., 2016; Helaly et al., 2017; Mir et al., 2022).

JA is a key hormonal molecule that regulates both plant development and defence mechanisms. It also works as a stress hormone, playing a crucial role in how plants respond to environmental challenges such as salinity, drought, temperature fluctuations, and heavy metal toxicity (Raza et al., 2022). SA is a vital hormone that acts as a key signalling molecule, regulating both plant immunity and growth (Rivas-San and Plasencia, 2011; Li et al., 2022). In this study, increases in both JA (all treatments) and SA levels (MiKS 2) (**Figure 5**) suggest an activation of defence pathways, with these hormones playing a key role in enhancing the plant’s response to both biotic and abiotic stresses. In a study conducted on grapevine cv. Cabernet Sauvignon, Wang et al. (2022) also reported an increase in both hormones following Kl application. Similarly, Jang et al. (2018) observed that Si application led to an increase in both hormones, further corroborating the findings of the present study.

Regarding the histological parameters (**Table 6**), all treated plants generally exhibited thicker upper and lower cuticles, a feature that may enhance disease resistance and reduce water loss (Ferrón-Carrillo and Urrestarazu, 2021). Interestingly, Brito et al. (2019) found no significant differences in upper cuticle thickness following Kl application. The differences observed in this study are likely attributed to the Si deposition induced by Si application. Previous research has shown a similar trend, where Si application resulted in increased cuticle thickness (Pozo et al., 2015; Ferrón-Carrillo and Urrestarazu, 2021). Therefore, Si application appears to reduce membrane permeability and transpiration through the cuticle, ultimately improving the plant’s water balance (Romero-Aranda et al., 2006), which is in accordance with the findings presented in the present study.

It is worth noting that the findings of this study were obtained using the red grape variety Touriga Franca. Therefore, it would be relevant to explore whether similar responses to the combined application of Si and Kl can be observed in other grape varieties, such as white ones. For instance, due to the differences in phenolic composition, cuticle thickness, and natural photoprotection, white cultivars may exhibit distinct physiological and biochemical responses under summer stress conditions. Future research could help clarify the broader applicability of this strategy across different grapevine types.

## 5 Conclusions

The combined application of Si and Kl, particularly through MiKS 2, MiKS 3, and MiKS 4 treatments, significantly enhanced grapevine ecophysiological and biochemical performance. The applied treatments improved water use efficiency, photosynthetic capacity, stress tolerance, and secondary metabolite production, while also inducing hormonal balance and anatomical adaptation, ultimately increasing plant resilience/adaptation to summer stress. The synergistic interaction between Si and Kl optimized physiological responses and can impact fruit quality, highlighting their potential as effective tools to enhance grapevine resilience in stressful environmental conditions. The present study also suggests that the integration of Si and Kl applications in vineyard management practices, particularly in drought-prone regions could be used as a beneficial cultural practice.

Considering all the parameters evaluated, MiKS 3 emerged as the most promising formulation, as it promoted significant improvements in plant performance while showing only minor differences when compared to MiKS 4. Therefore, the increased cost associated with the use of 8% Si in MiKS 4 may not be justified, given the similar outcomes achieved with the 6% Si concentration in MiKS 3.

Future research would consider the long-term impacts of these treatments on grape composition and wine quality across different grapevine types, including white varieties. Additionally, incorporating oxidative stress indicators such as total antioxidant capacity and malondialdehyde (MDA) levels would provide a more comprehensive understanding of the redox status and the protective mechanisms triggered by the combined application of Si and Kl under adverse climatic conditions.

## Data Availability

The original contributions presented in the study are included in the article/supplementary material. Further inquiries can be directed to the corresponding authors.
